# Ecological insights into the microbiology of food using metagenomics and its potential surveillance applications

**DOI:** 10.1099/mgen.0.001337

**Published:** 2025-01-03

**Authors:** Samuel J. Bloomfield, Falk Hildebrand, Aldert L. Zomer, Raphaëlle Palau, Alison E. Mather

**Affiliations:** 1 Quadram Institute Bioscience, Norwich Research Park, Norwich, UK; 2 Centre for Microbial Interactions, Norwich Research Park, Norwich, UK; 3 Earlham Institute, Norwich Research Park, Norwich, UK; 4 Utrecht University, Utrecht, Netherlands; 5 University of East Anglia, Norwich, UK

**Keywords:** antimicrobial resistance, food, metagenomes, pathogens

## Abstract

A diverse array of micro-organisms can be found on food, including those that are pathogenic or resistant to antimicrobial drugs. Metagenomics involves extracting and sequencing the DNA of all micro-organisms on a sample, and here, we used a combination of culture and culture-independent approaches to investigate the microbial ecology of food to assess the potential application of metagenomics for the microbial surveillance of food. We cultured common foodborne pathogens and other organisms including *Escherichia coli*, *Klebsiella/Raoultella* spp., *Salmonella* spp. and *Vibrio* spp. from five different food commodities and compared their genomes to the microbial communities obtained by metagenomic sequencing following host (food) DNA depletion. The microbial populations of retail food were found to be predominated by psychrotrophic bacteria, driven by the cool temperatures in which the food products are stored. Pathogens accounted for a small percentage of the food metagenome compared to the psychrotrophic bacteria, and cultured pathogens were inconsistently identified in the metagenome data. The microbial composition of food varied amongst different commodities, and metagenomics was able to classify the taxonomic origin of 59% of antimicrobial resistance genes (ARGs) found on food to the genus level, but it was unclear what percentage of ARGs were associated with mobile genetic elements and thus transferable to other bacteria. Metagenomics may be used to survey the ARG burden, composition and carriage on foods to which consumers are exposed. However, food metagenomics, even after depleting host DNA, inconsistently identifies pathogens without enrichment or further bait capture.

**Impact Statement**

Multiple micro-organisms can be found on food, including those that are dangerous to humans and those that carry antimicrobial resistance genes. Metagenomics involves analyzing all the microbial DNA on a sample, and in this study, we compared the metagenomes of food samples with the genomes of pathogens cultured from the same samples to determine if metagenomics could be used for food pathogen surveillance. Retail food samples were predominated by psychrotrophic bacteria that thrive in the cool temperatures at which food is stored. Pathogens accounted for a small percentage of the food metagenome compared to the psychrotrophic bacteria, and cultured pathogens were inconsistently identified in the metagenome data. For 59% of antimicrobial resistance genes (ARGs) found on food, metagenomics could predict what bacterial genus to which they belonged. However, it was unclear what percentage of ARGs could easily be transferred between bacteria. Metagenomics may be used to survey the burden of ARG on food but is currently unable to identify food pathogens effectively.

## Data Summary

All raw reads generated as part of this study were uploaded to the Sequence Read Archive (SRA) under the following projects: PRJNA849983 (food metagenome Illumina), PRJNA1034280 (chicken metagenome PromethION), PRJNA1107355 (leafy greens, pork and salmon metagenome PromethION), PRJNA939716 (*Salmonella* Illumina), PRJNA1135353 (*Klebsiella* Illumina) and PRJNA1107692 (*Escherichia coli* and *Vibrio* Illumina). The R scripts used to analyse food metagenomes were uploaded to Zenodo: doi.org/10.5281/zenodo.13995543.

## Introduction

Global trade provides the opportunity to consume food from around the world, originating from a wide range of animals and plants and produced using a range of methods. A safe food chain relies on the identification and minimization of food pathogens [1] and the identification of other micro-organisms that may indirectly influence a consumer’s health, such as antimicrobial-resistant (AMR) bacteria [2]. New molecular techniques are being developed to identify micro-organisms quickly, such as loop-mediated isothermal amplification to detect food pathogens [3], and at higher resolution, such as metagenomics that can identify micro-organisms at the strain level [4], but their effectiveness needs to be assessed before their utility for food surveillance can be determined.

It is estimated that foodborne pathogens are responsible for 2.4 million infections in the UK each year, including well-known bacterial pathogens such as *Campylobacter* spp., *Salmonella* spp. and *Listeria monocytogenes* [5]. However, not all individuals with foodborne illness will seek medical attention, and fewer still will submit a clinical sample for testing [6,7], preventing the causative agent and putative sources from being identified. Consumers are at risk when causative agents of foodborne illness remain undetected, typically due to traditional approaches being targeted to specific pathogens (e.g., culture-based, immunoassay and molecular methods) [1,8] or new foodborne diseases emerging and not being recognized [9]. As such, methods that can identify all micro-organisms on food in a timely, accurate and reproducible manner are required.

Pathogen identity is not the only important factor for food safety, as not all pathogens within the same species represent the same risk to human health. Pathogenic bacteria often contain virulence genes that encode for molecules that help to colonize, move between and evade host defenses, as well as obtain nutrients from their environment [10]; virulence genes are often used to distinguish pathogenic and non-pathogenic strains [11]. For this reason, multiple molecular methods have been developed to identify these virulence genes and the pathogenic strains carrying them [12].

The human burden of foodborne disease is further compounded by AMR. Although many foodborne illnesses are self-limiting, severe infections can occur that may require antimicrobial treatment, which is complicated by AMR. Pathogens and non-pathogenic bacteria on food can also act as a reservoir of antimicrobial resistance genes (ARGs) [13].

Beyond this, metal-tolerance genes are often co-selected with ARGs [14], with metals often used in the agricultural and horticultural industries for their bactericidal and growth-promoting effects [15,16]. Most studies of metal-tolerant bacteria on food focus on pathogens [17], although many of the non-pathogenic bacteria on food are known to be metal tolerant [18].

The gut microbiota consists of all the micro-organisms in the gastrointestinal tract and is influenced by diet [19]. The gut microbiota contain many metabolic pathways that can utilize and produce metabolites [20]. Collectively, the conserved sequences of reactions that encode for metabolite utilization or production are referred to as metabolic modules [21]. Food varies in chemical compositions, and certain macromolecules in food promote certain micro-organisms in the diet [19]. However, food may also influence the gut microbiota by introducing new strains that colonize the gut.

Previously, we developed a method for depleting host (food) DNA from food and applied it to 109 food samples [22]. In this study, for the 109 samples, we compared the genomes of cultured pathogens to the short-read metagenomes, supplementing a subset of samples with long-read metagenomes, with the purpose of obtaining a deeper understanding of the microbial ecology of foods and evaluating the usefulness of metagenomics in improving food surveillance and safety [23]. We also investigated microbial signatures amongst different food commodities and the ability of metagenomics to predict what bacteria contribute to the ARG reservoir on food.

## Methods

### Samples

The 109 food metagenomes analyzed in this study had previously undergone host DNA depletion before they were sequenced on an Illumina NextSeq [22]. They consisted of 28 chicken, 24 leafy green, 15 pork, 18 prawn and 24 salmon samples and were collected at retail throughout Norfolk County of the UK, as previously described [22]. The food samples were collected on 14 sampling trips, and a blank was run for each sampling trip that consisted of buffered peptone water that underwent the same host DNA depletion and sequencing was performed as for the food samples.

### Pathogen culturing

The food samples from which the metagenomes were extracted were also cultured for *Escherichia coli*, *Klebsiella/Raoultella* spp., *Salmonella* spp. and, in the case of seafood samples, *Vibrio* spp., as described previously [1]. Multiple isolates from each food sample were selected and analyzed further. For *E. coli*, up to four isolates per sample were analyzed; for *Salmonella*, up to eight isolates per sample were analyzed; and for *Klebsiella/Raoultella* and *Vibrio*, up to two isolates per sample were analyzed.

Genomes were extracted using the Maxwell RSC Cultured Cells DNA Kit (Promega, Madison, WI, USA). Libraries were created using the Nextera XT DNA Library Preparation Kit (Illumina, San Diego, CA, USA) and sequenced on a NextSeq 550 System (Illumina) as 150 bp paired-end reads.

Genomic Illumina reads were trimmed using fastp v0.19.5 [24] to trim reads with a minimum quality value of 20 and default other parameters. Trimmed genomic reads were assembled using SPAdes v3.11.1 [25] in ‘careful’ mode. The quality of the assemblies was assessed using QUAST v4.6.3 [26] and CheckM v1.1.2 [27] and by aligning reads to the assemblies using the Burrows–Wheeler aligner (BWA) v0.7.17 [28]. Assemblies were accepted if they consisted of less than 500 contigs that were over 500 bp and less than 50 duplicate genes and had a mean read depth of the 4 largest contigs above 30.

MLST v2.16.1 (https://github.com/tseemann/mlst) was used to predict the sequence types (STs) of the assembled genomes.

### Assembly-independent metagenomics

The raw paired Illumina reads of the 109 food samples and 14 blanks that were previously sequenced at 8 GB per metagenome [22] were processed using fastp similar to genomes. The blanks had been spiked with PhiX, and these reads were filtered out using BBsplit v38.75 [29] and the phiX174 genome (SAMN04281799).

MetaPhlAn v2.6.0 [30] was used to predict the taxonomic origin of the trimmed reads and to identify if reads associated with the cultured pathogens could be detected. In R v4.1.2 [31], phyloseq v1.38.0 (https://github.com/joey711/phyloseq) was used to rarefy the samples to the minimum sample total read depth, and ALDEx2 v1.26.0 (https://github.com/ggloor/ALDEx2_dev) was used to identify taxa associated with food commodity using a generalized linear model, and the level of significance was adjusted for multiple comparisons using the Benjamini–Hochberg procedure. A large percentage of the blanks’ reads were classified as *E. coli* reads, likely because the PhiX they were spiked with originated from *E. coli* and PhiX read filtering did not remove all of them.

MetaMLST v1.2.2 [32] was used to identify bacterial STs in food metagenomes using the paired trimmed reads. The STs identified in metagenomes were compared to the STs of pathogens cultured from the same samples.

To identify pathogen genomes within metagenomes, trimmed metagenome Illumina reads were aligned to the assembled genomes using BWA, and the coverage was calculated using SAMtools v1.9 [33]. For each genome, the percentage of the assembly to which metagenomic reads were aligned was calculated.

### Genome reconstruction and taxonomic and functional profiling of metagenomes

To investigate the differences in the food microbiota at the species level, MATAFILER was used to process metagenome raw reads, assemble metagenomes and reconstruct and dereplicate metagenome-assembled genomes (MAGs) [34]. Raw metagenomic reads were quality filtered using sdm v1.63 with default parameters [35]. Kraken2 [36] was used to remove metagenome reads associated with hosts using reference genomes of the organisms from which the food originated (Table S1, available in the online version of this article). Host-filtered metagenome reads were assembled using MEGAHIT v1.2.9 [37], and reads were back mapped to the assembly using Bowtie2 v2.3.4.1 [38]. Genes were predicted with Prodigal v2.6.1 with parameters ‘-p meta’ [39], and a gene catalogue clustered at 95% nt identity using MMseqs2 [40]. Matrix operations on the gene catalogue were carried out using rtk [41].

MAGs were calculated using SemiBin2 [42], and their completeness and contamination were estimated using CheckM2 [43]. Using a combination of genome bins (via canopy clusters [44] as implemented in canopy2, https://github.com/hildebra/canopy2) and SemiBin2 MAGs, high-quality MAGs (>80% completeness and <5% contamination) [34] were dereplicated using clusterMAGs (https://github.com/hildebra/clusterMAGs). The GTDB bacterial core genes were used to guide dereplication. The dereplication procedure uses a canopy clustering approach, requiring at least (weighted) >80% matching in marker genes between two MAGs to collapse into the same species. These dereplicated MAGs are referred to as metagenome species (MGSs). MGSs associated with individual food commodities were identified based on the relative abundance of the MGSs after rarefaction using ALDEx2 as previously described.

To identify functional groups that differed between metagenomes, gene annotation was performed on the metagenome assemblies as described by Frioux *et al.* [45]. Briefly, the gene catalogue was annotated to the eggNOG database [46] using the BLASTPmodule of Diamond [47]. Higher-level Kyoto Encyclopedia of Genes and Genomes (KEGG) Orthology (KO) categories were downloaded from the KEGG database [48]. The KO assignments were used to predict conserved sequences of reactions that make up metabolic modules represented using GOmixer [49,50]. ALDEx2 was used to determine if food commodity was associated with the relative abundance of metabolic modules after rarefaction as described previously.

KMA v1.2.3t [51] was used to identify ARGs using the ResFinder v3.2 database [52] and virulence genes using the Virulence Finder Database (VFDB) v6.0 [53]. Lapidary v0.5.0 [54] was used to identify metal-tolerance genes using the BacMet database [55]. For AMR, virulence and metal-tolerance genes, 90% identity and 60% coverage cut-offs were used. The number of reads that aligned to these genes was compared to the number of post-host DNA-removed reads. phyloseq was used to rarefy the samples to the minimum sample post-host DNA-removed reads. ALDEx2 was used to look for an association between the genes of interest and food commodity as described previously.

### Read depth and pathogen identification comparison

A linear regression model was used to determine if the number of sequenced microbial reads or relative abundance of pathogen was associated with the detection of pathogens. The outcome variable was the percentage of the pathogen genome assembly that reads from the associated metagenome aligned to using BWA, and the explanatory variables were the number of microbial reads (post-Kraken2 host removal), relative abundance of the specific pathogen (calculated by MetaPhlAn2) and the pathogen identity. A partial F-test was used to determine if pathogen identity significantly affected the model.

### Long-read analysis

For the 24 food metagenomes that had a DNA concentration above 30 ng µl^−1^, libraries were formed using the Ligation Sequencing Kit (SQK-LSK110) (Oxford Nanopore Technologies, Oxford, UK) and barcoded using Native Barcoding Expansion 1–12 (EXP-NBD104) and 13–24 (EXP-NBD114) (Oxford Nanopore Technologies). These libraries were sequenced on a PromethION (Oxford Nanopore Technologies) at a target depth of 8 GB per metagenome. Bases were called using Guppy v6.2.1 (https://nanoporetech.com/document/Guppy-protocol) with super accuracy basecalling. The raw long reads were trimmed using NanoFilt v0.1.0 (https://github.com/wdecoster/nanofilt). Hybrid assemblies with trimmed long and short reads were assembled using OPERA-MS v0.9.0 [56].

ARGs, virulence genes and plasmid replicons were identified in hybrid assemblies using Abricate v1.01 (https://github.com/tseemann/abricate) with the ResFinder v3.6, VFDB v2.0.4 and PlasmidFinder v2.1.3 [57] databases, respectively. Metal-tolerance genes were identified using TBLASTN v2.14.0 [58] and the BacMet v2.0 database. For Abricate and TBLASTN, 90% identity and 60% coverage cut-offs were used. For ARG-, metal-tolerance- and virulence-containing contigs, the gene of interest was blocked out and the taxonomic origin of the contigs was predicted using Kraken v2.1.1 [36] and the maxikraken2_1903_140 GB databases. ISEscan v1.7.2.3 [59] was used to identify insertion sequences (ISs), and those classified as ‘complete’ were investigated further. Genes of interest within 10 kbp of ISs were classified as associated with ISs.

The percentage of the metagenome comprised of each gene of interest was calculated by multiplying the gene length by the mean Illumina read depth of the contigs on which the genes were located and dividing by the number of post-host DNA-removed sequenced bases of the metagenome.

To measure the accuracy of contig-based gene taxonomic classifications, the analysis was repeated on the genomes of the bacteria cultured from the same samples as the metagenomes, including masking the genes of interest, except with 90% identity and coverage cut-offs. The bacterial genomes sequenced in this study consisted of well-described pathogens, whilst the metagenomes contained many poorly described micro-organisms. A lower coverage cut-off was used when identifying genes of interest in metagenomes (60%) compared to genomes in case the poorly described pathogens contained genes distantly related to those in the gene databases and would be missed with a stricter coverage cut-off [60].

## Results

### Metagenome-genome comparisons

**Fig. 1.**
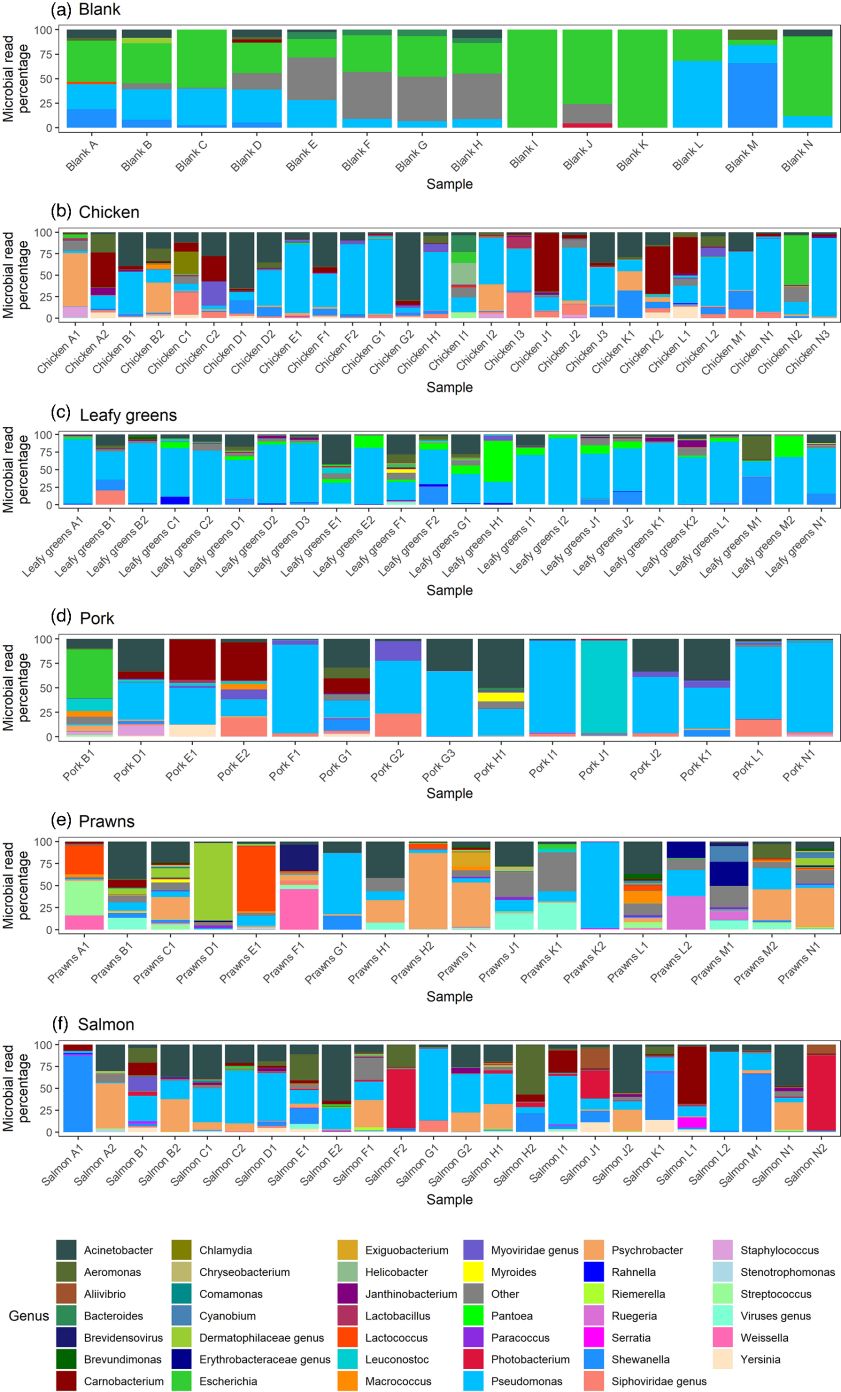
Barplots of the microbial reads classified at the genus level for the blanks (**a**), chicken (**b**), leafy green (**c**), pork (**d**), prawn (**e**) and salmon (**f**) samples for 109 short-read sequenced metagenomes and 14 blanks. The top 40 most common genera were coloured, and the rest were combined into an ‘other’ category.

Analyses of the metagenomic reads from food samples using MetaPhlAn2 revealed a large amount of variation in the microbial composition of food (Fig. 1); the most abundant genera were *Pseudomonas* (39%), *Acinetobacter* (14%), *Psychrobacter* (6.8%), *Shewanella* (5.6%) and *Carnobacterium* (5.0%). For each sampling run, different batches of buffered peptone water and other reagents were used as sampling took place over a 7-month period, explaining the differences in the microbial composition of the blanks.

To test the ability of metagenomics to accurately detect pathogens on food, we cultured specific pathogenic bacteria from the 109 food samples. Culturing results varied amongst different food commodities (Table S2), as described in previous work [1]. Of the 109 food samples tested, 62% were positive for *E. coli*, 31% for *Klebsiella/Raoultella* and 2.8% for *Salmonella* through culture. Of the 42 seafood samples tested, 26% were positive for *Vibrio* through culture. For 98% of metagenomes, the relative abundance of pathogens was less than 10% compared to the other micro-organisms in the metagenome, regardless of whether or not the pathogen was recovered from the sample through culture. The exception was a chicken and a pork sample, where the relative abundance of *E. coli* was 49% and 27 %, respectively. *E. coli* was cultured from these samples (Fig. S1).

The ability of metagenomes to detect low-abundant pathogens was measured by investigating if MetaPhlAn2 found any reads of the pathogens cultured from food. When compared to culture results, MetaPhlAn2 was 0–38% sensitive and 78–100% specific for the different pathogens investigated (Table S3).

After removing host reads from the food metagenomes, three metagenomes (one chicken and two prawn samples) had too few reads for subsequent assemblies. These samples were excluded from further analysis.

**Fig. 2.**
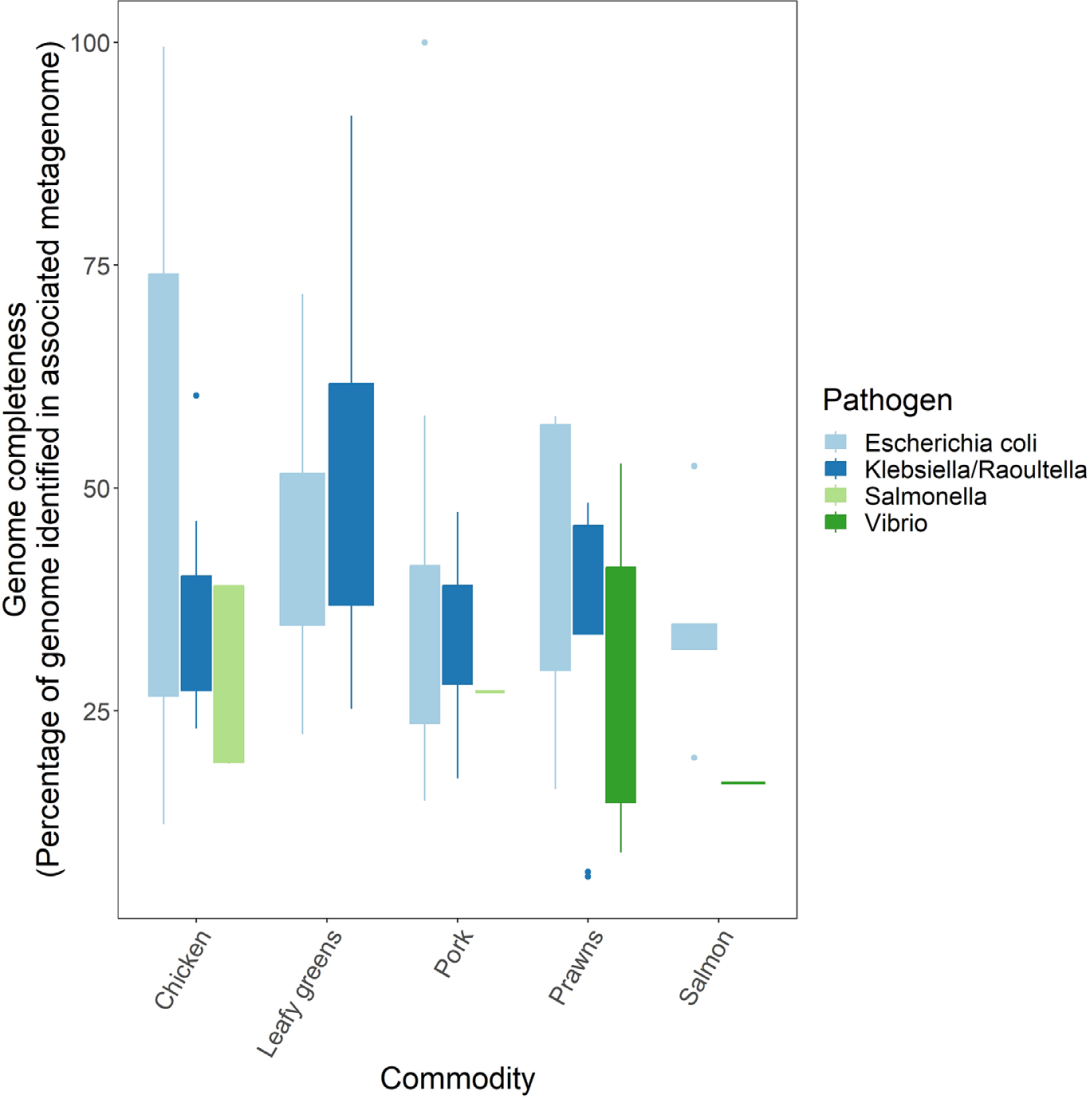
Percentage of genome assemblies of pathogens cultured from food identified by the associated short-read sequenced metagenome, separated by food commodity and coloured by taxa.

For the isolated pathogens, the associated metagenomes aligned to 6.3–99.9% of their genome assemblies (Fig. 2). The percentage of the genome assembly identified was positively associated with the number of microbial reads sequenced (linear regression: *P*=3.3×10^−4^) and the relative abundance of the specific pathogen compared to other micro-organisms in the metagenome (linear regression: *P*=7.3×10^−16^). Pathogen identity was also associated with the percentage of the genome assembly identified (partial F-test: *P*=0.011). The pork metagenome consisting of 27% *E. coli* microbial reads had three *E. coli* isolates cultured from it that belonged to ST 2967, and 99.9% of their assemblies were identified via metagenomic sequencing. The chicken sample consisting of 49% *E. coli* microbial reads had three *E. coli* isolates cultured from it that belonged to different STs, 57, 131 and 8611, and 89.5–99.6% of their assemblies were identified in the metagenome. For the rest of the pathogens cultured from food, 6–98% of their genome assemblies were found in the associated metagenome.

MetaMLST was used to identify STs in the metagenomes and was able to identify STs of at least 1 bacterial species in 21 of the 109 food samples analyzed (Table S4). However, most of the STs were unknown, with only three known STs identified. The only known pathogen ST identified was *E. coli* ST 353. This was identified in the chicken metagenome where this species comprised 49% of the microbial reads, but none of the *E. coli* isolates cultured from this sample belonged to this ST.

### Metagenomic microbial composition and metabolic modules

Metagenomic sequences were further processed using MATAFILER [34] to reconstruct *de novo* MGSs associated with food commodity. MGS analysis identified 111 taxa at the species level across the 106 food metagenomes with sufficient host-removed reads to assemble that were each represented by at least one high-quality reference MAG (>80% completeness and <5% contamination). MGS relative abundance analysis using ALDEx2 identified five taxa that differed between food commodities. Compared to other food commodities, leafy greens were associated with a higher relative abundance of *Pantoea agglomerans*; chicken and pork were associated with a higher relative abundance of *Brochothrix thermosphacta*, *Pseudomonas paraversuta* and *Pseudomonas weihenstephanensis*; and chicken, pork and salmon were associated with a higher relative abundance of *Acinetobacter harbinensis* (Fig. S3). Using non-metric multidimensional scaling, there was some overlap amongst chicken, pork, prawn and salmon samples, whilst leafy green samples clustered separately to the other food commodities (Fig. S4). Additional taxa were associated with food commodity when reads were taxonomically classified using MetaPhlAn, although MetaPhlAn struggled to classify some taxa at the species level (Fig. S5).

**Fig. 3.**
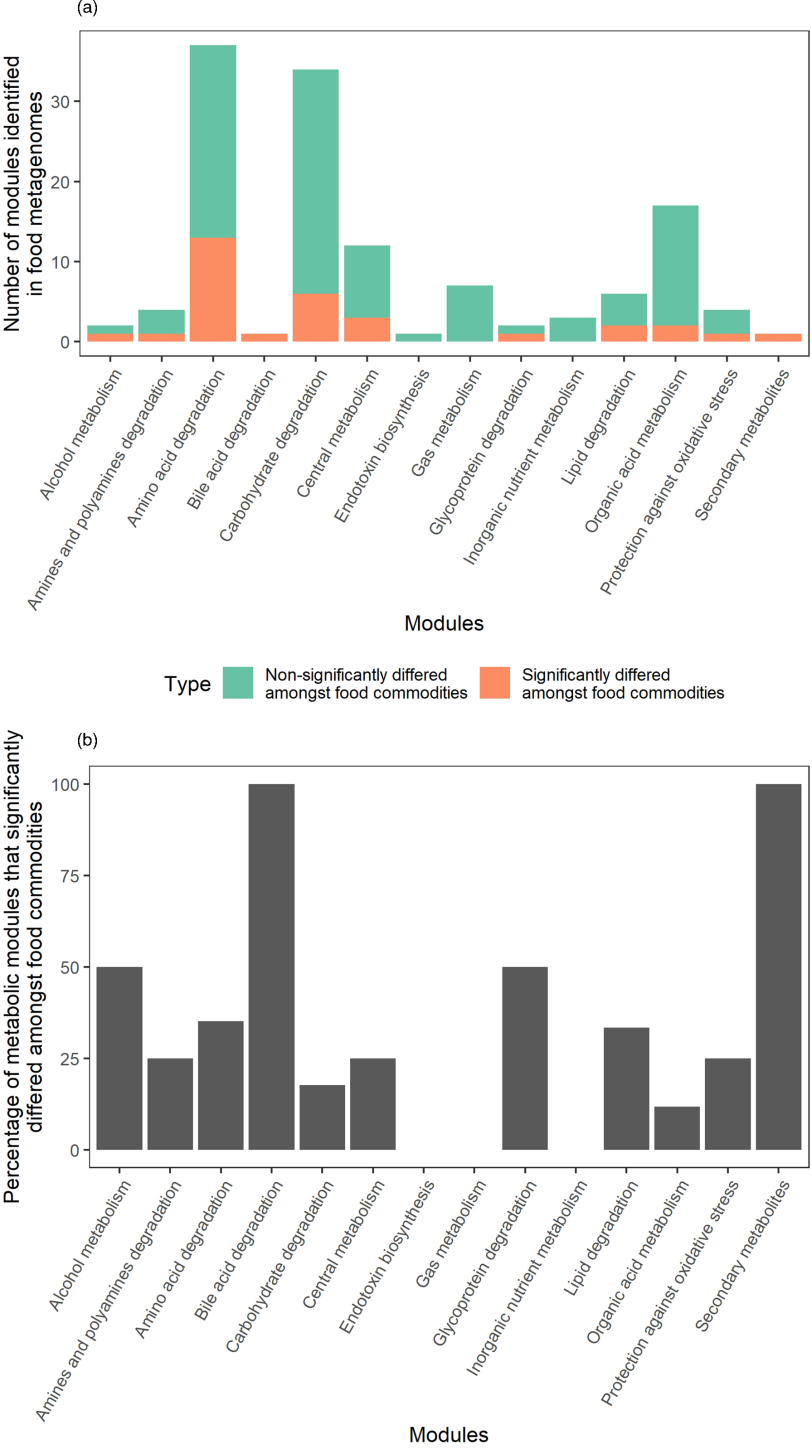
Number (**a**) and percentage (**b**) of metabolic modules identified amongst 106 short-read sequenced food metagenomes whose assemblies passed quality control and that significantly differed between samples from different food commodities.

Metabolic analysis identified 131 metabolic modules in the food metagenomes. Thirty-two modules significantly differed between food commodities (Fig. S6). The 131 metabolic modules were classified into 14 functional groups, and the metabolic modules that differed amongst food commodities were not associated with functional group (Fisher’s exact test: *P*=0.183) (Fig. 3). Leafy green samples had a higher relative abundance of pectin degradation compared to other food commodities.

ARG analysis identified 368 ARGs across the food metagenomes. Comparisons of ARG concentration within the food metagenome identified three that significantly differed between food commodities (Fig. S7). *blaERP-1* was associated with a higher concentration in leafy greens; *tet(L)* in chicken and pork; and *tet(39)* in chicken, pork and salmon.

Virulence gene analysis identified 922 virulence genes across the food metagenomes. Five virulence genes significantly differed between food commodities: *mrkA*, *mrkB*, *mrkC*, *mrkD* and *mrkF* were associated with a higher concentration in chicken, pork and salmon samples (Fig. S8). These genes are associated with bacterial fimbriae and biofilm formation. The VFDB associates each virulence gene with a specific pathogen; for each pathogen cultured, the culture status was compared with the detection of one or more virulence genes associated with that pathogen. When compared to culture results, virulence gene detection was 0–71% sensitive and 11–94% specific for the pathogens investigated (Table S5).

Metal-tolerance gene analysis identified 413 metal-tolerance genes across the food metagenomes. A total of 125 metal-tolerance genes significantly differed between food commodities, of which 65 were associated with tolerance to multiple compounds (Fig. S9). These genes were associated with tolerance to 61 compounds, most commonly with tolerance to mercury (Fig. S10).

### Long-read metagenomes

Long-read sequencing was applied to 24 metagenomes to improve metagenome assembly length and help taxonomically investigate ARGs, along with metal-tolerance and virulence genes. Long-read sequencing was applied to 7 chicken, 11 leafy green, 5 pork and 1 salmon samples. Hybrid long- and short-read assembled metagenomes had a mean N50 value of 17 981 bp (range: 3760–41 102 bp) and a mean largest contig of 1 258 817 bp (range: 365 137–4 020 681 bp).

**Fig. 4.**
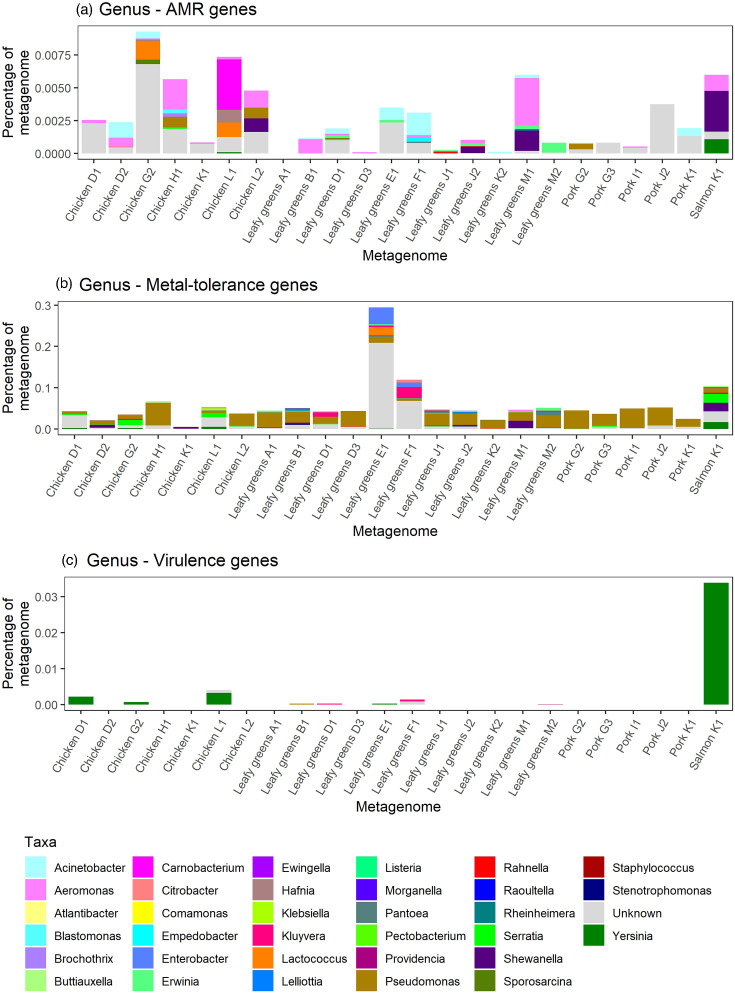
Percentage of metagenomes comprised of antimicrobial resistance (AMR) (**a**), metal-tolerance (**b**) and virulence (**c**) genes identified in 24 long-read metagenome assemblies and coloured by the predicted taxa at the genus level.

ARGs comprised 0–0.009% of the metagenome, metal-tolerance genes 0.005–0.3% of the metagenome and virulence genes 0–0.03% of metagenomes (Fig. 4). The taxonomic origin was predicted for 59% of AMR, 68% of metal-tolerance and 96% of virulence genes at the genus level, but greater percentages were predicted at higher taxon levels (Fig. S11). The genera most commonly found with ARGs were *Aeromonas* (18%), *Shewanella* (9.4%), *Acinetobacter* (9.2%) and *Carnobacterium* (6.0%). Amongst the 24 hybrid metagenomes, 66 unique ARGs were identified and 35 were identified in multiple contigs. Of those identified multiple times, eight were consistently found with the same genus and four were found with different genera (Fig. S12). The genus most commonly found with metal-tolerance genes was *Pseudomonas* (41%), and that most commonly found with virulence genes was *Yersinia* (93%).

**Fig. 5.**
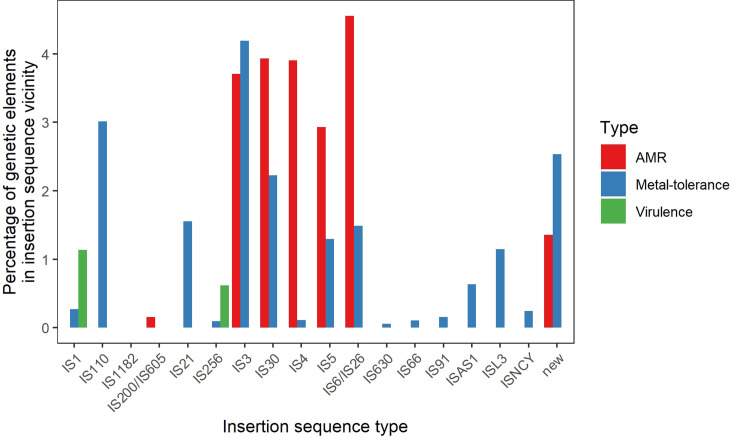
Percentage of AMR, metal-tolerance and virulence genes in the presence of different insertion sequence types for the 24 long-read food metagenomes.

Amongst the 24 hybrid assembled metagenomes, 173–1303 (mean=680) ISs and 0–47 (mean=12) plasmid replicons were identified. Using a 10 000 bp cut-off, 16% (sample range: 0–49%) of ARGs were associated with ISs, as were 13% (sample range: 0–77%) of metal-tolerance genes and 1.8% (sample range: 0–100%) of virulence genes. ARGs were most commonly associated with IS3 (3.7%; sample range: 0–39%), IS4 (3.9%; sample range: 0–25%), IS5 (2.9 %; sample range: 0–36%), IS6/IS26 (4.6%; sample range: 0–15%) and IS30 (3.9%; sample range: 0–17%) (Fig. 5). 6.0 % (sample range: 0–52%) of ARGs, 0.07% of metal-tolerance genes (sample range: 0–0.77%) and no virulence genes were found on the same contig as a plasmid replicon.

**Fig. 6.**
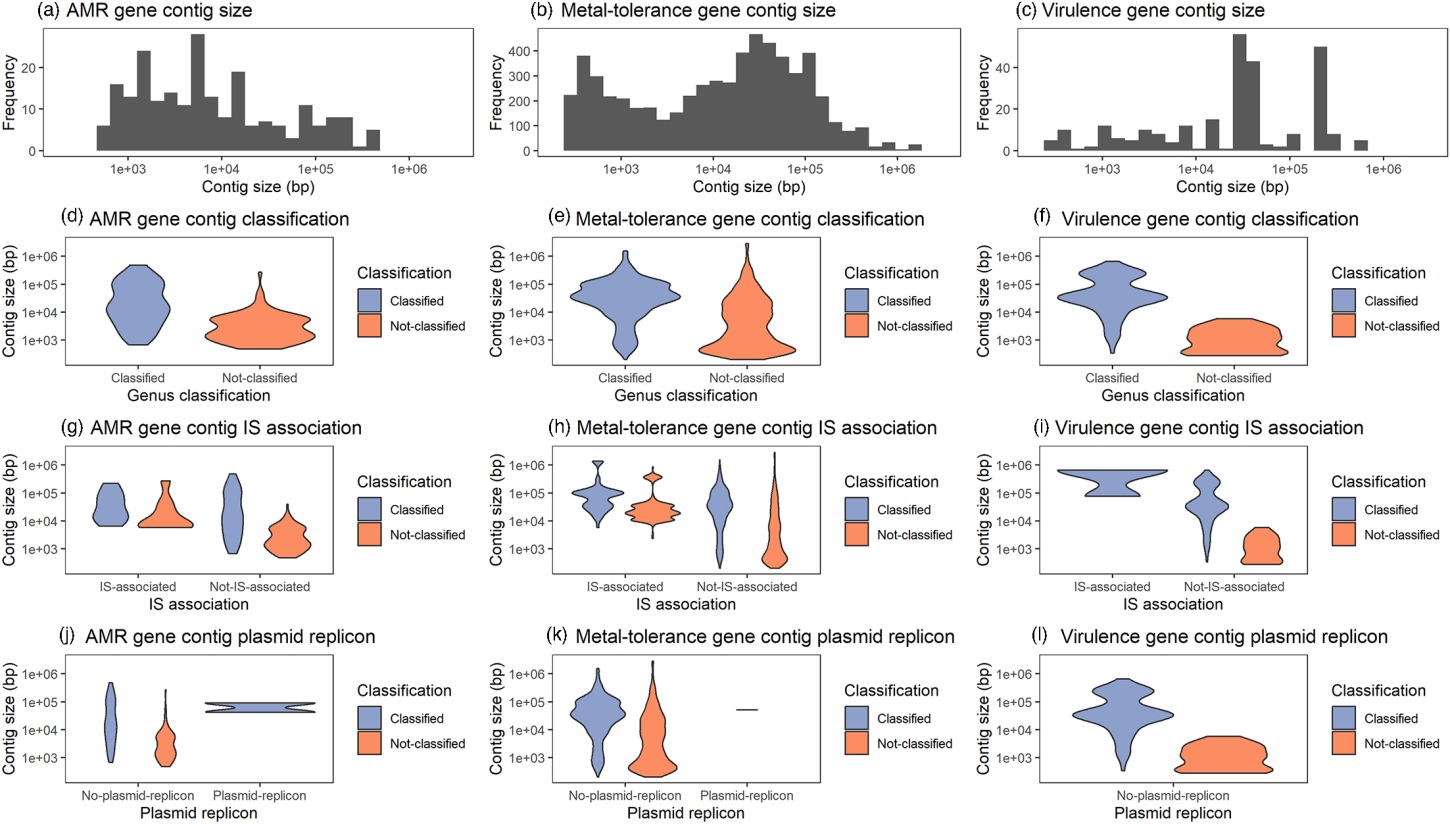
Size distribution of antimicrobial resistance (AMR) (**a, d, g, j**), metal-tolerance (**b, e, h, k**) and virulence (**c, f, i, l**) gene-containing contigs (**a–c**) for 24 long-read sequenced food metagenomes, separated by whether they were classified at the genus level (**d–f**), contained insertion sequences (ISs) (**g–i**) or contained plasmid replicons (**j–l**).

The ARG-containing contigs were 479–485 710 bp (mean: 37 227 bp) in length, metal-tolerance gene-containing contigs were 202–2 849 978 bp (mean: 65 141 bp) in length and virulence gene-containing contigs were 273–665 242 bp (mean: 85 980 bp) in length (Fig. 6). Above 10 000 bp, the origin of most contigs was predicted at the genus level. There was no association between taxonomic classification at the genus level and being associated with an IS for ARG-containing (Fisher’s exact test: *P*=0.53), metal-tolerance-containing (Fisher’s exact test: *P*=0.18) or virulence-containing (Fisher’s exact test: *P*=1.0) contigs.

To validate the taxonomic classification of metagenomic elements of interest, the analysis was repeated on the cultured isolate genomes where the taxonomy was known. None of the genome contigs containing AMR, metal-tolerance or virulence genes were misclassified, but only 17% of ARGs, 11% of metal-tolerance genes and 12% of virulence genes were classified at the genus level (Figs S13–S15). However, the percentage of genes classified at the genus level differed between the pathogens cultured for the genes of interest investigated (Fisher’s exact text: *P*<1×10^−8^, 1×10^−8^ and 1×10^−8^, respectively), and larger percentages of genes were classified at higher taxon levels.

## Discussion

Metagenomics has the potential to revolutionize food safety diagnostics, allowing for the identification and typing of pathogens and tracking AMR in a matter of hours or at most days [23]. However, by comparing the genomes of pathogens cultured from food samples to the metagenomic detection of the same pathogens, we demonstrate that it is currently not practical in all scenarios. Its ability to detect the presence of pathogens or genetic traits, which may be in low abundance, must be evaluated before it should be applied to food surveillance.

A wide range of micro-organisms were found on food that varied in abundance, but the predominating micro-organisms belonged to the *Acinetobacter*, *Carnobacterium*, *Pseudomonas*, *Psychrobacter* and *Shewanella* genera. These bacteria are mostly psychrotrophic, able to replicate on food when refrigerated. We also investigated the ability of metagenomics to detect the presence of particular bacteria relevant to food safety (*E. coli*, *Klebsiella/Raoultella*, *Salmonella* and *Vibrio*), whose presence was confirmed through a culture-based approach. A pork and a chicken sample had a large percentage of *E. coli* reads in their metagenome, and from the *E. coli* cultured from these samples, we were able to identify >99% of the genomes of the *E. coli* cultured from the pork sample and >99% of the genome of one of the *E. coli* isolate genomes cultured from the chicken sample. The chicken sample had a diverse population of *E. coli* present, as indicated by the multiple *E. coli* STs we cultured from the sample, and our ST analysis of the metagenomic reads indicated that we did not isolate the most prominent *E. coli* ST. Therefore, if there is a diverse population of a bacterial species, then metagenomics will likely identify the most prominent strain, which may be missed if culturing methodologies involve an enrichment step and isolating a small number of colonies. However, for the remaining samples, the cultured pathogens comprised less than 10% of the micro-organism reads on food, and the percentage of their genomes identified in metagenomes varied from 6 to 98%. In addition, investigating low-abundant pathogens in metagenomes by the presence of any reads classified as belonging to pathogens was not a very sensitive approach when compared to culture results, nor was searching metagenomes for virulence genes associated with pathogens. Therefore, our results indicate that efficient identification and metagenomic characterization of pathogens on food require either (a) deeper metagenomic sequencing or (b) specific enrichment of pathogens or their DNA.

The number of microbial reads sequenced in a food metagenome is dependent on the overall sequencing depth and the amount of host DNA removed prior to sequencing. The food metagenomes analysed in this study had undergone host DNA depletion, but the method used worked better for certain food commodities over others [22], leaving variation in the number of microbial reads. The percentage of the pathogen genomes identified in the associated metagenomes was related to the number of microbial reads sequenced. However, the linear regression results indicate that 4.1×10^6^ additional microbial reads need to be sequenced in the metagenome to increase the percentage of pathogen assembly in the metagenome by 1%. Therefore, the read depth would need to be magnitudes higher to ensure the sensitive identification of pathogens on food. Previously, shotgun metagenomics was used to detect *Brucella* in host DNA-depleted raw milk samples [61]. However, the raw food commodities in our study likely have higher concentrations of non-pathogenic micro-organisms than raw milk, explaining its inability to detect culturable pathogens in many samples. Leonard *et al.* [62] were able to detect Shiga toxin-producing *E. coli* on spinach, but only after an 8-h enrichment that significantly altered the microbiota. Therefore, on food samples with a large concentration of non-pathogens present, pathogen enrichment by growing the pathogens or rejecting host DNA through approaches such as Oxford Nanopore adaptive sequencing [63] is required. However, both enrichment approaches will alter the relative abundance of micro-organisms in the microbiota, preventing analyses that rely on abundance.

Virulence genes encode microbial components that are often required for pathogens to cause infections [10]. The short-read analysis identified 922 virulence genes in the food metagenomes, but only 5 significantly differed amongst food commodities, which were associated with chicken, pork and salmon. These virulence genes are associated with *Klebsiella pneumoniae* in the VFDB but have been isolated from other bacteria, such as *Pseudomonas* from food [64]. For 24 metagenomes, we also had long-read sequences supplementing the short-read sequences. Most virulence genes in these samples were associated with *Yersinia*, in concordance with our previous study that demonstrated the presence of *Yersinia enterocolitica* on 76–80% of meat and seafood samples [65], but almost all isolates belonged to the non-pathogenic biotype 1A. These virulence genes could also belong to other *Yersinia* species, such as *Yersinia intermedia* or *Yersinia ruckeri* for which we identified MGSs in some of the food metagenomes, but no human clinical infections have been found associated with these species [66].

Pathogens are not the only risk to humans on food, as food can also carry micro-organisms that are resistant to antimicrobial drugs. Hybrid metagenome analysis predicted that most ARGs which could be associated with specific bacterial hosts originated from potential opportunistic pathogens (e.g. *Acinetobacter* and *Aeromonas*) and environmental organisms (e.g. *Carnobacterium* and *Shewanella*). However, there were a large number of ARGs whose taxonomic origin we could not predict, and when the approach was applied to the genomes of cultured bacteria, there was a lot of variation between the percentage of genes classified and the pathogen cultured, suggesting that this approach may be better at predicting the origin of ARGs belonging to some taxa compared to others. ARG databases are also biased towards culturable and/or pathogenic bacteria [67], and these databases need to be extended to be relevant to other bacterial species. The taxonomic predictions also do not take into consideration if ARGs are transferable to other bacteria, which is relevant for the assessment of potential risk to human health.

One way to evaluate the potential mobility of ARGs is to assess their proximity to ISs. These genetic elements facilitate the movement of genes around and between genomes, including ARGs [68]. In the 24 metagenomes which had long-read sequences in addition to short-read sequences, 16% of ARGs were associated with ISs and the most common IS types were IS3, IS4, IS5, IS6/IS26 and IS30. Most of these IS types have been found to be highly or variably associated with ARGs in bacterial pathogens and in animal and environmental metagenomes [69]. In addition, 6% of ARGs were found on plasmid replicon-containing contigs. The small number of ARGs associated with ISs and plasmids could suggest that only a small percentage of the ARGs on food are transmissible to other bacteria but is more likely due to the fragmented metagenome assemblies. Even with long-read sequencing, metagenomic analysis could not assemble all chromosomes and plasmids into single contigs, leaving many of the ARGs on small contigs, and neither their bacterial host nor genomic location (chromosome, plasmid or other mobile genetic element) could be determined. However, even if all plasmids were assembled into single contigs, we may still struggle to identify the origin of the plasmid if it is associated with a wide range of bacteria. High-throughput chromosome conformation capture techniques crosslink sections of DNA that interact with each other before sequencing, allowing the identification of chromosomes and plasmids from the same cell [70], and may be required for plasmid taxonomic classification on food. When looking at specific ARGs, we identified some that were unlikely to be mobile, such as the *blaA* and *vatF* genes, which were predicted to be from *Yersinia* and are found in the chromosome of this bacterial genus [71,72]. However, four ARGs were associated with multiple bacterial genera and therefore likely to be mobile. Overall, this suggests that at least 32% (range: 0–100% amongst food samples) of ARGs in the food metagenome are mobile, as they are either associated with an IS, plasmid replicon or multiple genera. However, there was also a sample bias in this analysis, as long-read sequencing requires a higher DNA concentration than short-read sequencing, preventing long-read sequencing of samples with low DNA concentrations, as was observed for most seafood samples examined in this study [22].

Non-pathogenic bacteria on food are not just potential AMR carriers; they may also be beneficial to human health [73]. There is a large amount of variation in the nutrients and macromolecules found in food [74], which likely leads to the selection of micro-organisms that contain metabolic modules that allow them to utilize these nutrients and macromolecules. Most of the foods analyzed here are likely to be cooked prior to consumption, limiting the exposure of consumers to the micro-organisms identified. However, for leafy greens, there is likely to be little processing, and diets rich in leafy greens are likely to expose consumers to micro-organisms associated with specific carbohydrate degradation modules, such as pectin degradation. Pectins are polysaccharides found in many fruits and vegetables that are indigestible by human enzymes [75] but can be fermented in the gastrointestinal tract into products such as short-chain fatty acid (SCFA) [76], mostly in the large intestine [77]. Along with being a type of dietary fibre, pectins promote gastrointestinal health by promoting pectinolytic micro-organisms that can ferment pectin [78] and the immune-modulatory role of the SCFA produced [79]. Leafy greens may therefore be beneficial to the human diet by providing consumers with pectin and the micro-organisms to utilize them. Further work is required to identify the micro-organisms responsible for these modules on food and if they can colonize the gastrointestinal tract.

Outside of health risks and benefits, micro-organisms on food can also cause spoilage [80]. *Pseudomonas* is one of the main microbial causes of food spoilage [81] and is the predominant bacterial genus found on retail food [22]. Previously, we tested 32 food samples for *Pseudomonas*, including 8 that had their metagenomes sequenced and were included as part of this study, and identified a diverse population of this genus on food [64]. Metal-tolerance genes were most commonly associated with *Pseudomonas*, but this is likely because of how prevalent *Pseudomonas* is on food. In this study, we identified *Pseudomonas* species significantly differed between food commodities. Future work is required to determine how food spoilage alters the populations of different *Pseudomonas* species on food.

Consumer health relies on safe food. The microbial communities on retail food are predominated by psychrotrophic bacteria that benefit from the cool temperatures at which food is stored. Therefore, consistent identification of pathogens that are not adapted to thrive in cold conditions and thus comprise a small percentage of the overall microbial community on food will require methods that can enrich relevant bacteria and/or selectively sequence their genetic elements. The microbial composition of food varies amongst different commodities, and food metagenomics can predict the taxonomic origin of many ARGs on food at the genus level, but further work is required to determine what percentage of the ARGs on food is associated with mobile genetic elements and thus transferable to other bacteria. Food metagenomics could then be used to investigate what AMR bacteria consumers are exposed to on food, but currently, it is unable to consistently identify pathogens without enrichment.

## supplementary material

10.1099/mgen.0.001337 Uncited Supplementary Material 1.

## Abbreviations

AMR: antimicrobial resistance
ARG: antimicrobial resistance gene
BWA: Burrows–Wheeler aligner
ISs: insertion sequences
KEGG: Kyoto Encyclopedia of Genes and Genomes
KO: Kyoto Encyclopedia of Genes and Genomes Orthology
MAG: metagenome-assembled genome
MGSs: metagenome species
SCFA: short-chain fatty acid
ST: sequence type
VFDB: Virulence Finder Database
