# Supplementary material for: Ecological insights into the microbiology of food using metagenomics and its potential surveillance applications
Source: Microb Genom. 2025 Jan 3;11(1):001337. doi: 10.1099/mgen.0.001337 (PMC11893277; doi:10.1099/mgen.0.001337)
Supplement: Uncited Supplementary Material 1. [file mgen-11-01337-s001.pdf]

Supplementary materials

Supplementary table 1. Reference genomes used to filter out host reads

Commodity	Species	Reference
**Blank**	PhiX	NC 001422
**Chicken**	*Gallus gallus* 1	SAMN15960293
**Chicken**	*Gallus gallus* 2	SAMN20335985
**Chicken**	*Gallus gallus* 3	SAMN20335984
**Chicken**	*Gallus gallus* 4	SAMN20335982
**Leafy greens**	*Lactuca sativa*	SAMN01730979
**Leafy greens**	*Eruca vesicaria* subsp. *sativa*	SAMEA13191861
**Leafy greens**	*Spinacia oleracea*	SAMN05242642
**Leafy greens**	*Beta vulgaris*	SAMN07736104
**Leafy greens**	*Nasturtium officinale*	SAMEA4640853
**Leafy greens**	*Brassica rapa*	SAMN17245291
**Leafy greens**	*Lonicera japonica*	SAMN24662184
**Pork**	*Sus scrofa* 1	SAMN02953785
**Pork**	*Sus scrofa* 2	SAMN09531794
**Pork**	*Sus scrofa domesticus*	SAMN14543579
**Prawns**	*Penaeus monodon*	SAMN14330694
**Prawns**	*Penaeus vannamei*	SAMN08721527
**Salmon**	*Salmo salar*	SAMEA8062739
**Salmon**	*Oncorhynchus gorbuscha*	SAMN18987060
**Salmon**	*Oncorhynchus keta*	SAMN12367893
**Salmon**	*Oncorhynchus nerka*	SAMN11519041

Supplementary table 2. Percentage of each food commodity that were positive for pathogens through culture and the number of isolates sequenced

Bacteria	Commodity	Percentage positive	Isolates sequenced
Escherichia coli	Chicken	100%	109
	Leafy greens	75%	54
	Pork	93%	42
	Prawns	17%	8
	Salmon	21%	18
Klebsiella/Raoultella	Chicken	32%	13
	Leafy greens	63%	26
	Pork	40%	10
	Prawns	22%	8
	Salmon	0%	0
Salmonella	Chicken	7%	7
	Leafy greens	0%	0
	Pork	7%	4
	Prawns	0%	0
	Salmon	0%	0
Vibrio	Prawns	56%	15
	Salmon	4%	1

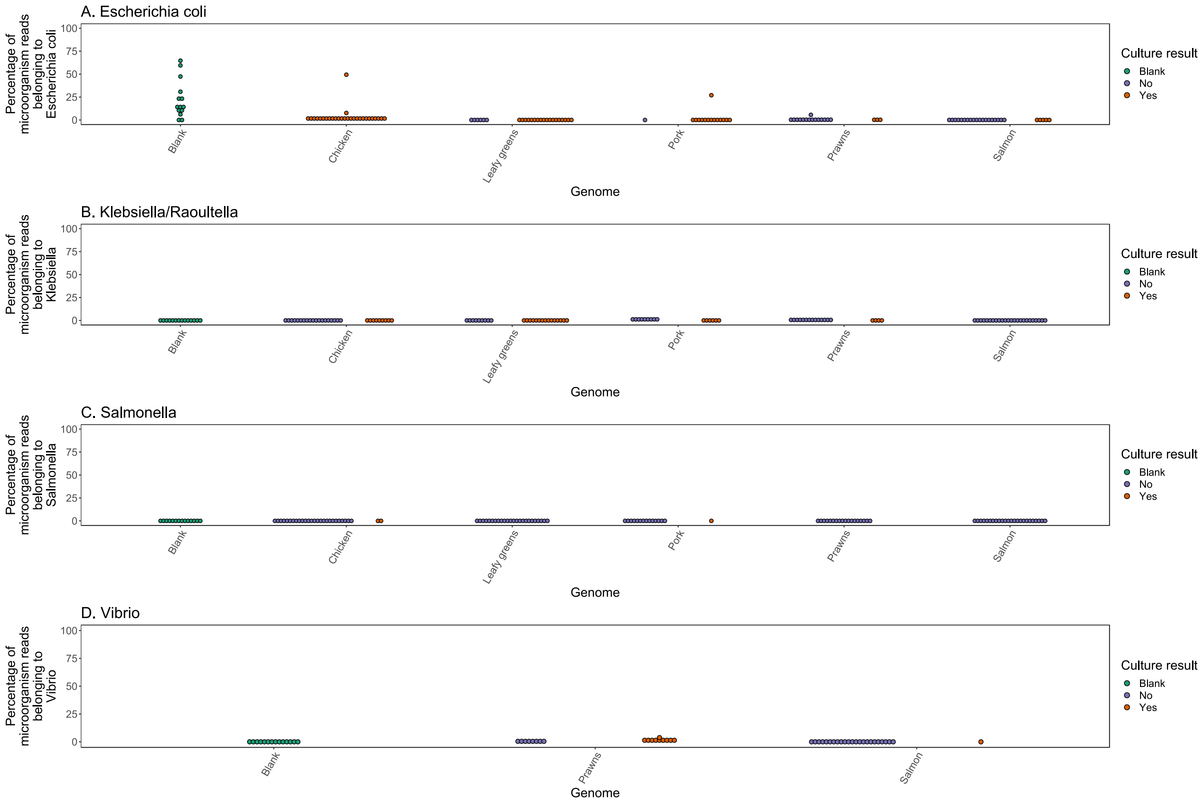

Supplementary figure 1. Percentage of food metagenome microorganism reads made up of *Escherichia coli* (A), *Klebsiella* (B), *Salmonella* (C) and *Vibrio* (D), and coloured by whether or not the samples were positive for the bacteria through culture for 109 short-read sequenced food metagenomes and 14 blanks.

Supplementary table 3. Sensitivity and specificity of Metaphlan2 for detecting pathogens cultured from the associated sample.

	Culture positive		Culture negative		Statistics	
Pathogen	Read positive	Read negative	Read positive	Read negative	Sensitivity	Specificity
Escherichia coli	26	42	9	32	38%	78%
Klebsiella	12	22	13	62	35%	83%
Salmonella	0	3	0	106	0%	100%
Vibrio	3	8	5	26	27%	84%

Supplementary table 4. Sequence types (STs) identified in metagenomes using metaMLST

Sample	STs
**Blank A**	
**Blank B**	
**Blank C**	
**Blank D**	
**Blank E**	
**Blank F**	
**Blank G**	
**Blank H**	
**Blank I**	
**Blank J**	
**Blank K**	
**Blank L**	
**Blank M**	
**Blank N**	
**Chicken A1**	
**Chicken A2**	
**Chicken B1**	*Carnobacterium maltaromaticum* (Unknown ST)
**Chicken B2**	*Carnobacterium maltaromaticum* (Unknown ST)
**Chicken C1**	*Yersinia pseudotuberculosis* (Unknown ST)
**Chicken C2**	*Carnobacterium maltaromaticum* (Unknown ST)
**Chicken D1**	
**Chicken D2**	
**Chicken E1**	
**Chicken F1**	*Carnobacterium maltaromaticum* (Unknown ST)
**Chicken F2**	
**Chicken G1**	
**Chicken G2**	*Carnobacterium maltaromaticum* (Unknown ST)
**Chicken H1**	
**Chicken I1**	*Streptococcus dysgalactiae* (249)
**Chicken I2**	*Pseudomonas fluorescens* (Unknown ST)
**Chicken I3**	
**Chicken J1**	
**Chicken J2**	
**Chicken J3**	*Carnobacterium maltaromaticum* (Unknown ST)
**Chicken K1**	
**Chicken K2**	*Carnobacterium maltaromaticum* (Unknown ST)
**Chicken L1**	
**Chicken L2**	
**Chicken M1**	
**Chicken N1**	
**Chicken N2**	*Campylobacter jejuni* (Unknown ST); *Escherichia coli* (353)
**Chicken N3**	*Carnobacterium maltaromaticum* (Unknown ST)

**Leafy greens A1**	
**Leafy greens B1**	
**Leafy greens B2**	
**Leafy greens C1**	
**Leafy greens C2**	
**Leafy greens D1**	
**Leafy greens D2**	
**Leafy greens D3**	
**Leafy greens E1**	
**Leafy greens E2**	
**Leafy greens F1**	
**Leafy greens F2**	
**Leafy greens G1**	
**Leafy greens H1**	
**Leafy greens I1**	
**Leafy greens I2**	
**Leafy greens J1**	
**Leafy greens J2**	
**Leafy greens K1**	
**Leafy greens K2**	
**Leafy greens L1**	
**Leafy greens M1**	
**Leafy greens M2**	
**Leafy greens N1**	
**Pork B1**	
**Pork D1**	*Carnobacterium maltaromaticum* (Unknown ST)
**Pork E1**	*Carnobacterium maltaromaticum* (Unknown ST); *Yersinia pseudotuberculosis* (Unknown ST); *Yersinia ruckeri* (Unknown ST)
**Pork E2**	
**Pork F1**	
**Pork G1**	
**Pork G2**	*Pseudomonas fluorescens* (Unknown ST)
**Pork G3**	
**Pork H1**	
**Pork I1**	
**Pork J1**	
**Pork J2**	
**Pork K1**	
**Pork L1**	
**Pork N1**	
**Prawns A1**	
**Prawns B1**	
**Prawns C1**	
**Prawns D1**	
**Prawns E1**	

**Prawns F1**	
**Prawns G1**	
**Prawns H1**	
**Prawns H2**	
**Prawns I1**	
**Prawns J1**	
**Prawns K1**	
**Prawns K2**	
**Prawns L1**	
**Prawns L2**	
**Prawns M1**	
**Prawns M2**	
**Prawns N1**	
**Salmon A1**	*Carnobacterium maltaromaticum* (Unknown ST)
**Salmon A2**	
**Salmon B1**	
**Salmon B2**	
**Salmon C1**	
**Salmon C2**	
**Salmon D1**	
**Salmon E1**	
**Salmon E2**	
**Salmon F1**	
**Salmon F2**	
**Salmon G1**	*Pseudomonas fluorescens* (Unknown ST)
**Salmon G2**	
**Salmon H1**	
**Salmon H2**	*Aeromonas* (Unknown ST); *Carnobacterium maltaromaticum* (Unknown ST)
**Salmon I1**	*Pseudomonas fluorescens* (Unknown ST)
**Salmon J1**	*Yersinia pseudotuberculosis* (396)
**Salmon J2**	
**Salmon K1**	
**Salmon L1**	*Carnobacterium maltaromaticum* (Unknown ST)
**Salmon L2**	
**Salmon M1**	
**Salmon N1**	
**Salmon N2**	

Pathogen assembly metagenome coverage linear regression

Residuals:

Minimum	First quartile	Median	Third quartile	Maximum
-34.9	-12.3	-3.58	8.97	51.3

Coefficients:

Coefficient	Estimate	Standard error	t-value	p-value
Intercept	29.8	3.48	8.56	6.13×10^{-16}
Microbial reads	2.44×10^{-7}	6.72×10^{-8}	3.64	3.26×10^{-4}
Relative abundance	1.67	0.196	8.53	7.27×10^{-16}
Pathogen: *Klebsiella*/*Raoultella*	1.57	2.84	0.552	0.581
Pathogen: *Salmonella*	-11.0	5.83	-1.88	6.13×10^{-2}
Pathogen: *Vibrio*	-14.9	5.48	-2.73	6.81×10^{-3}

Statistics:

Statistic	Value
Residual standard error	18.9 on 300 degrees of freedom
Multiple R^2	0.244
Adjusted R^2	0.231
F-statistic	19.4 on 5 and 300 degrees of freedom
p-value	$<2.2 \times 10^{-16}$

Pathogen identity pathogen genome assembly coverage partial-F test:

Model	Residual degrees of freedom	Residual sum of squares	Degrees of freedom	Sum of squares	F-value	p-value
Model with pathogen identity	300	106,548				
Model without pathogen identity	303	110,591	-3	-4,043	3.80	1.07×10^{-2}

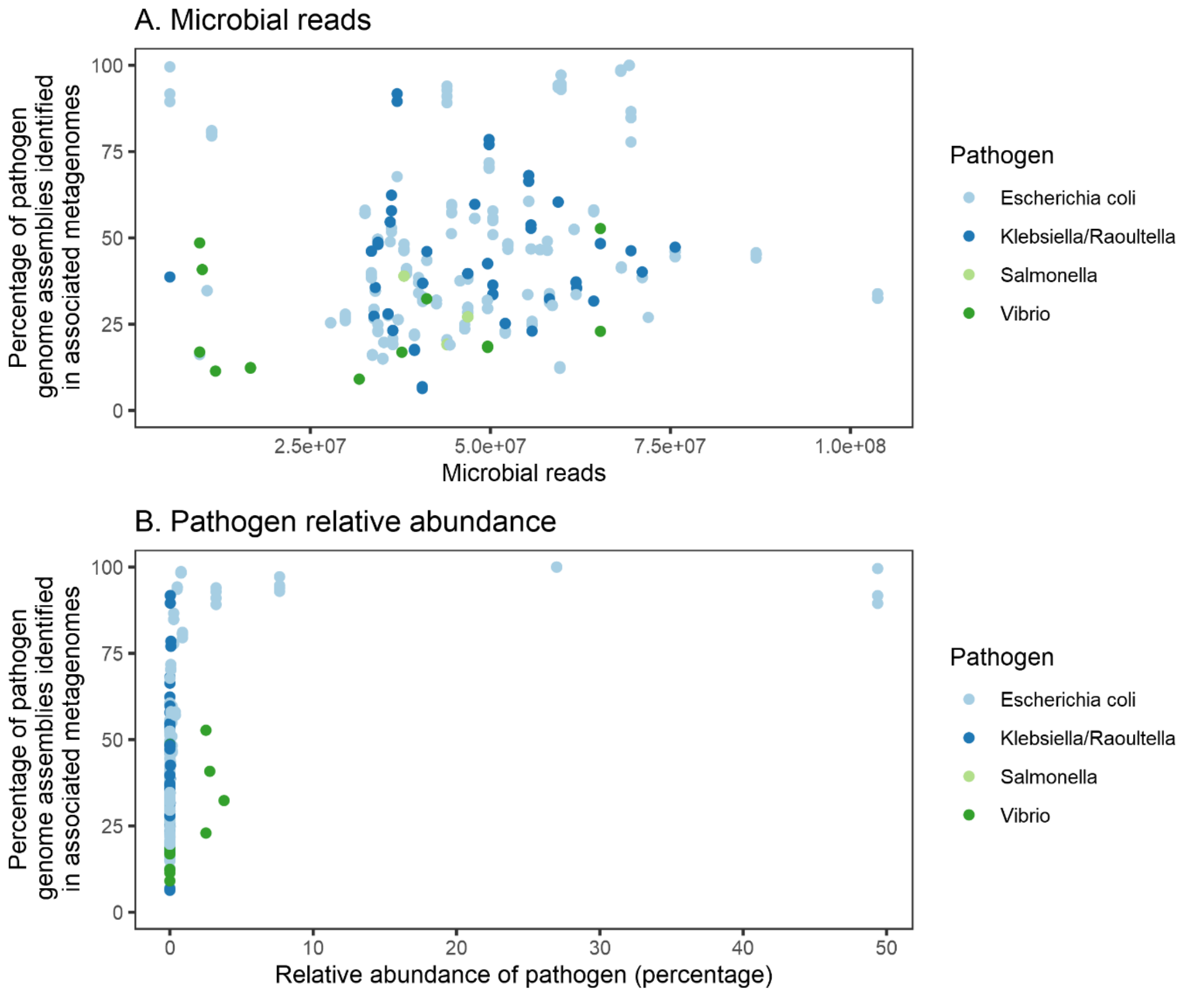

Supplementary figure 2. Percentage of genome assemblies identified in associated metagenomes versus the number of microbial reads sequenced (A) and the relative abundance of the pathogen in the metagenome (B); coloured by the pathogen analysed.

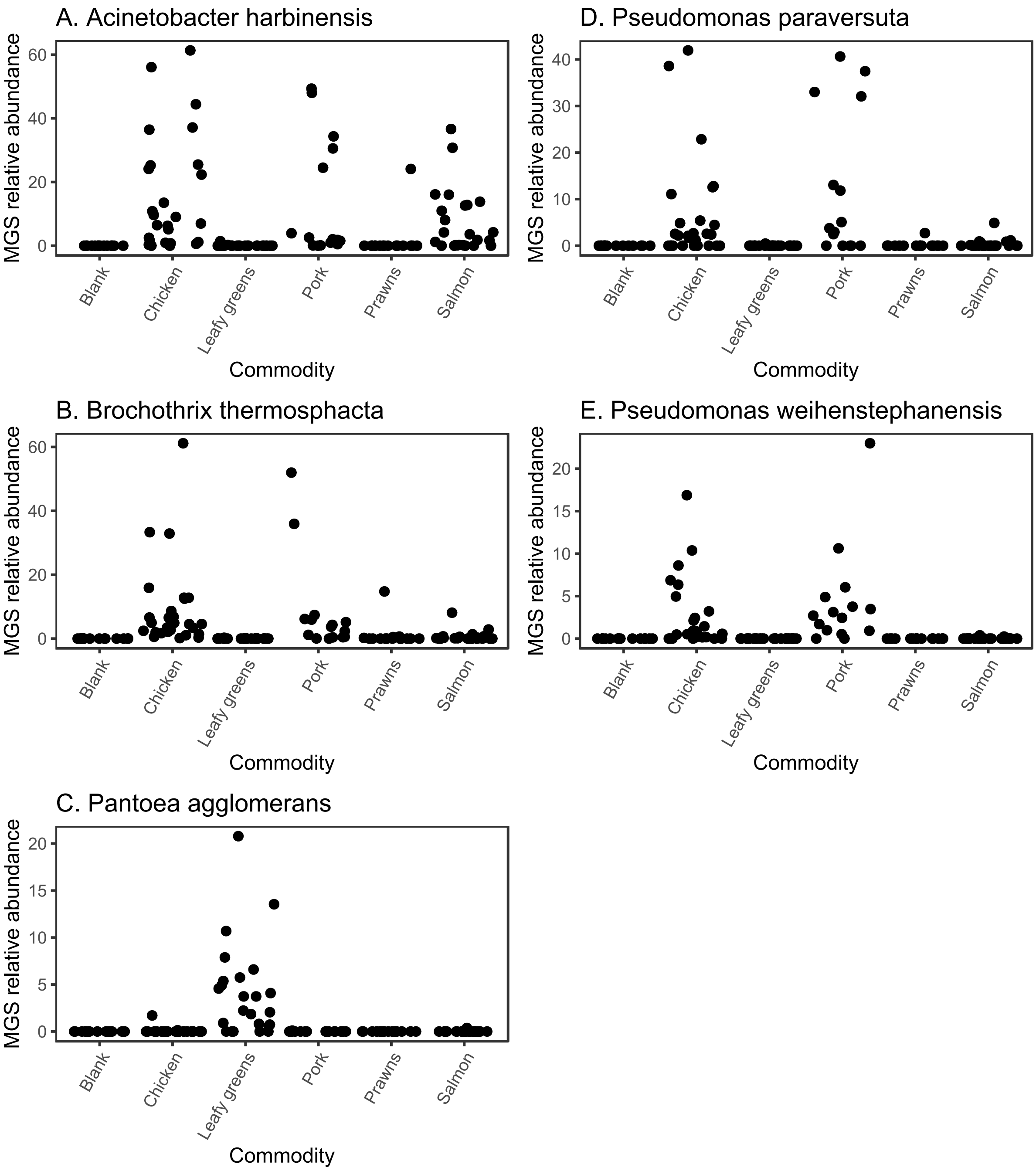

Supplementary figure 3. Metagenome signature (MGS) relative abundance for different food commodities that differed significantly amongst commodities amongst 106 short-read food metagenomes whose assemblies passed QC, and 14 blanks.

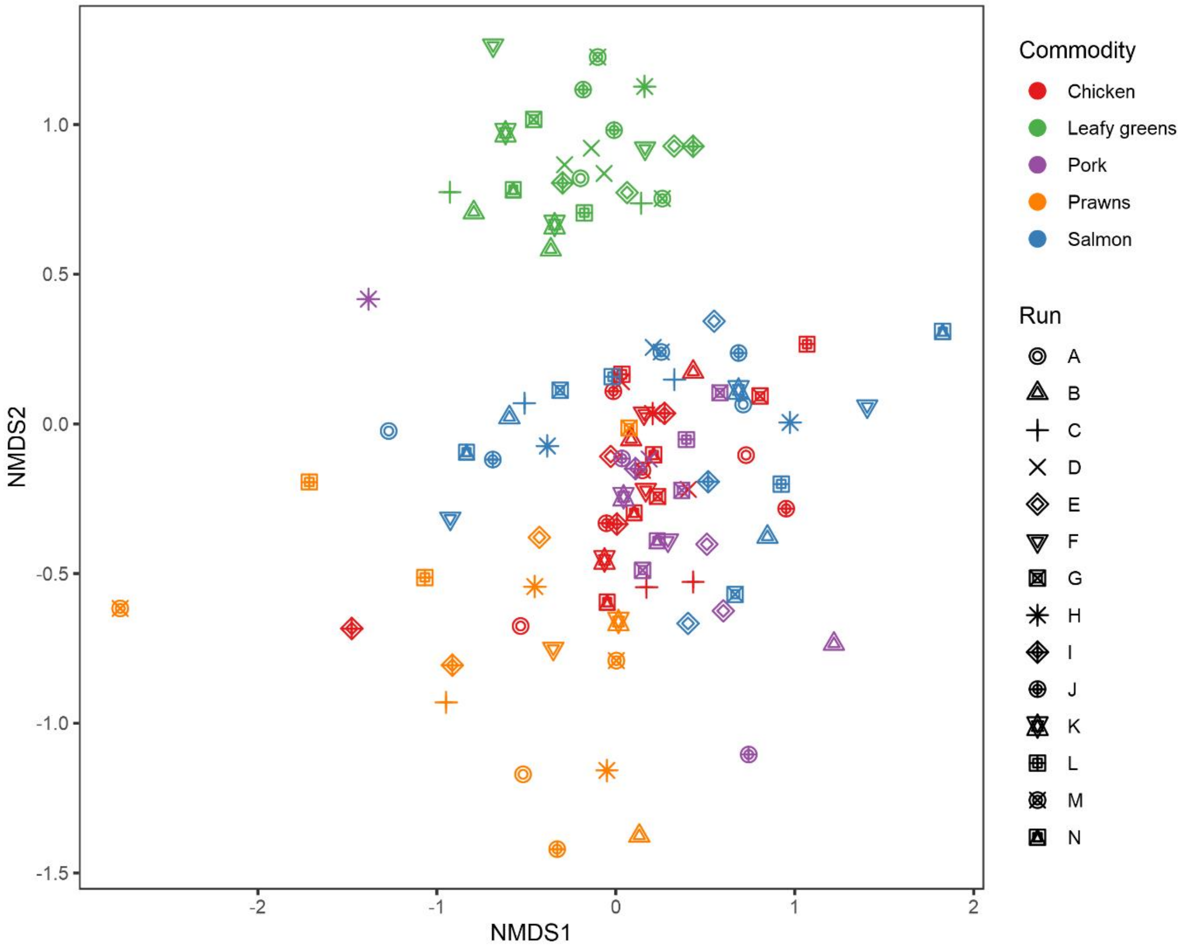

Supplementary figure 4. Non-metric multidimensional scaling of 106 short-read food metagenomes whose assemblies passed QC based on rarefied relative abundance of metagenome signatures (MGS), coloured by commodity and shaped by sampling runs.

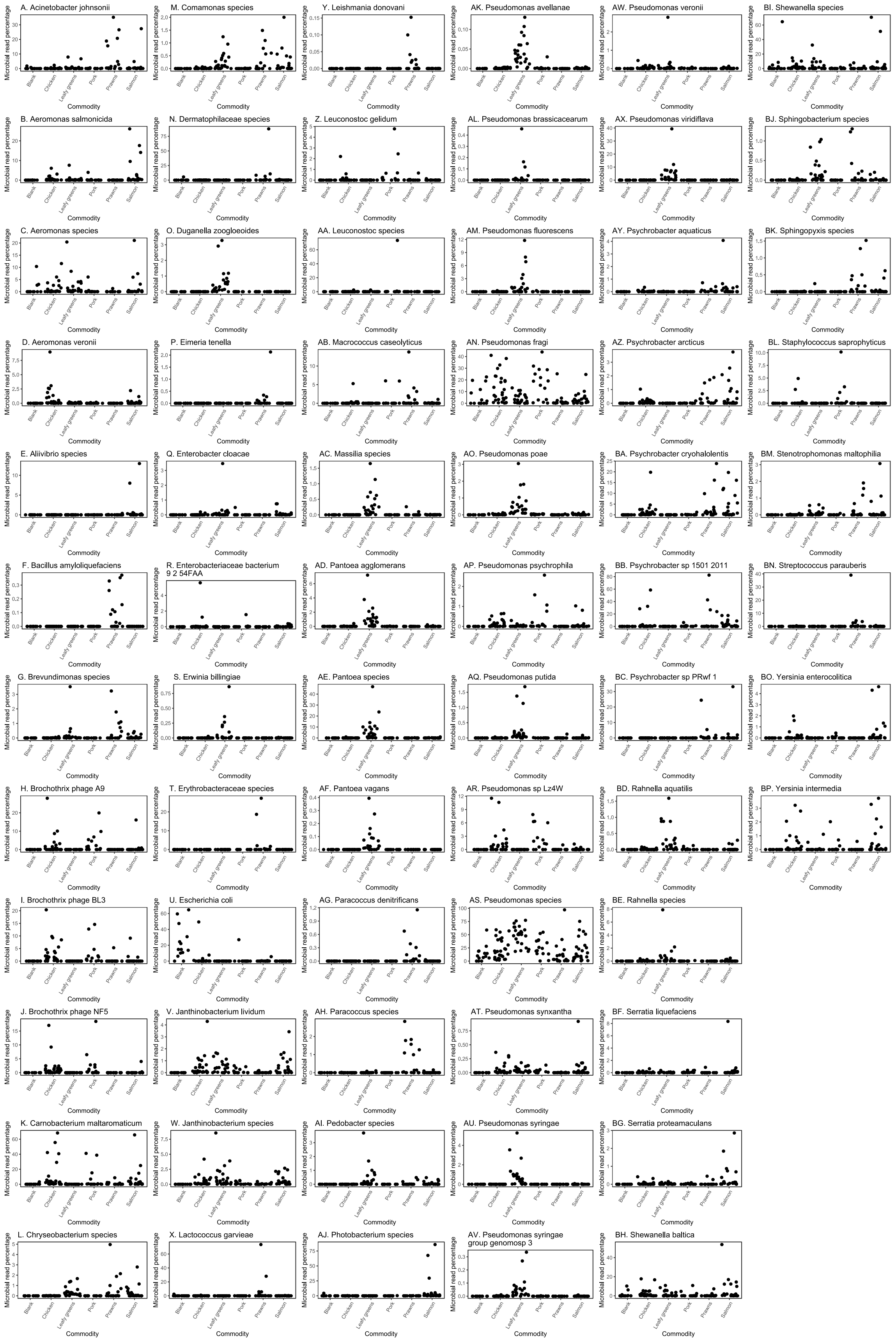

Supplementary figure 5. Metaphlan2 microorganism read percentage for different taxa that significantly differed between food commodities for 109 short-read sequenced food metagenomes.

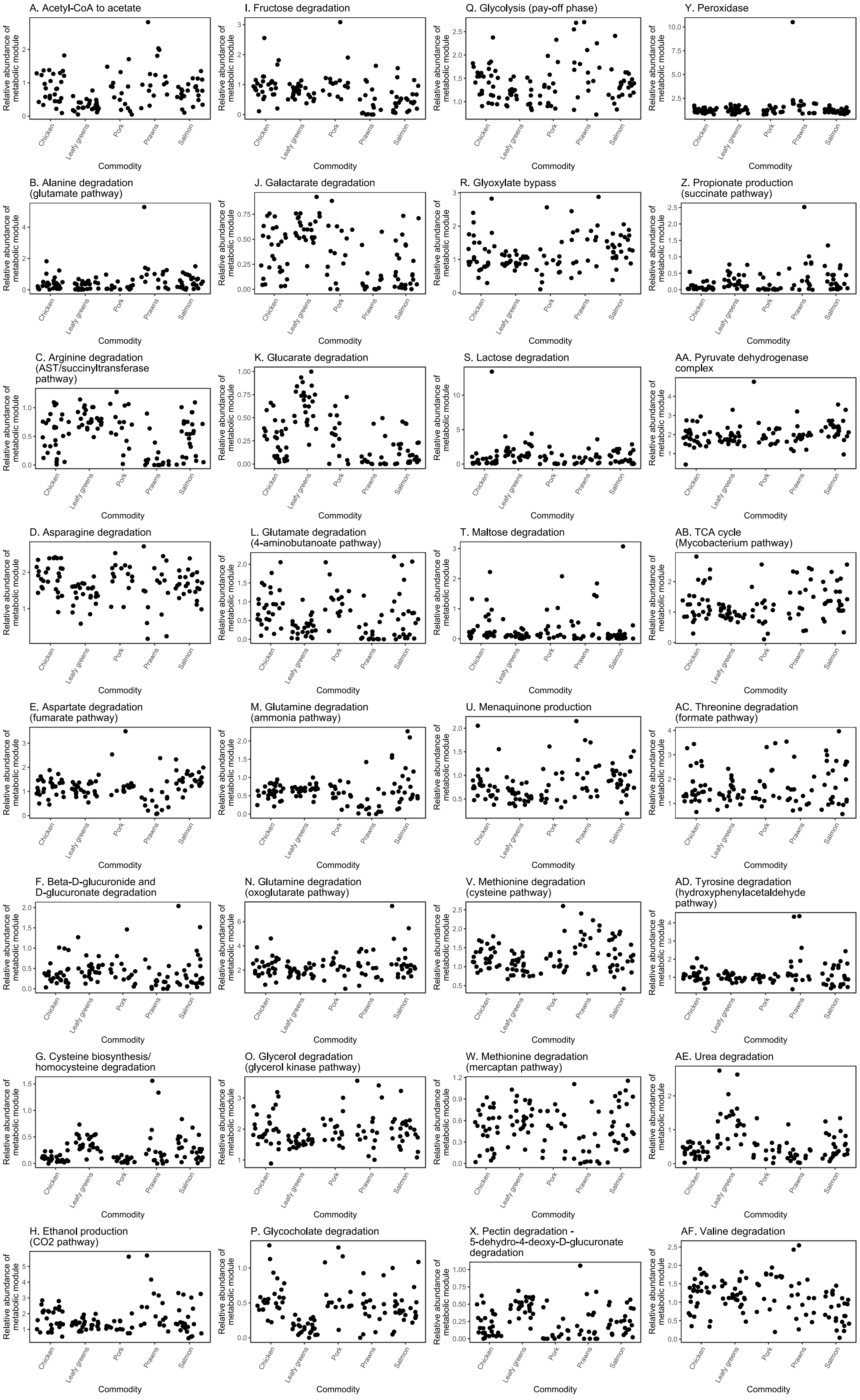

Supplementary figure 6. Relative abundance of metabolic modules that differed significantly amongst commodities for 106 short-read sequenced food metagenomes whose assemblies passed QC.

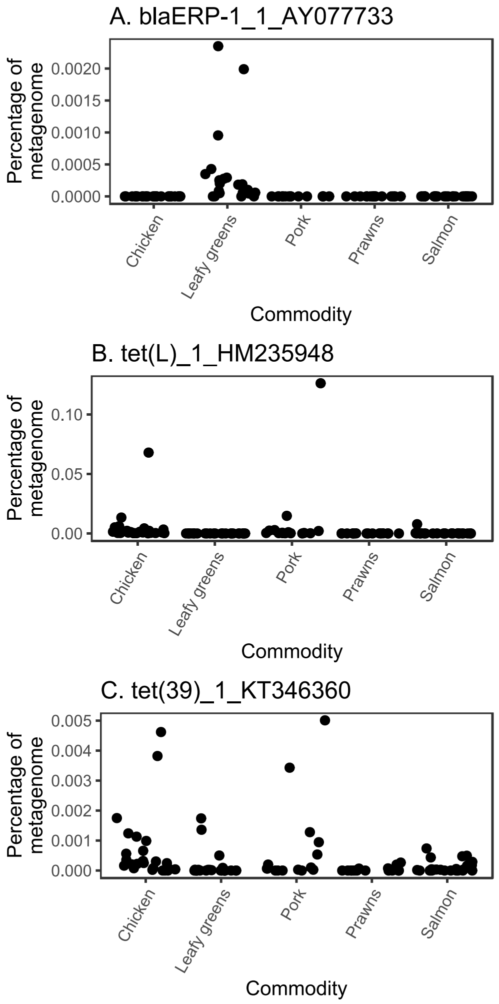

Supplementary figure 7. Percentage of host-removed reads made up of antimicrobial resistance genes that significantly differed amongst food commodities for 106 short-read sequenced food metagenomes that passed QC.

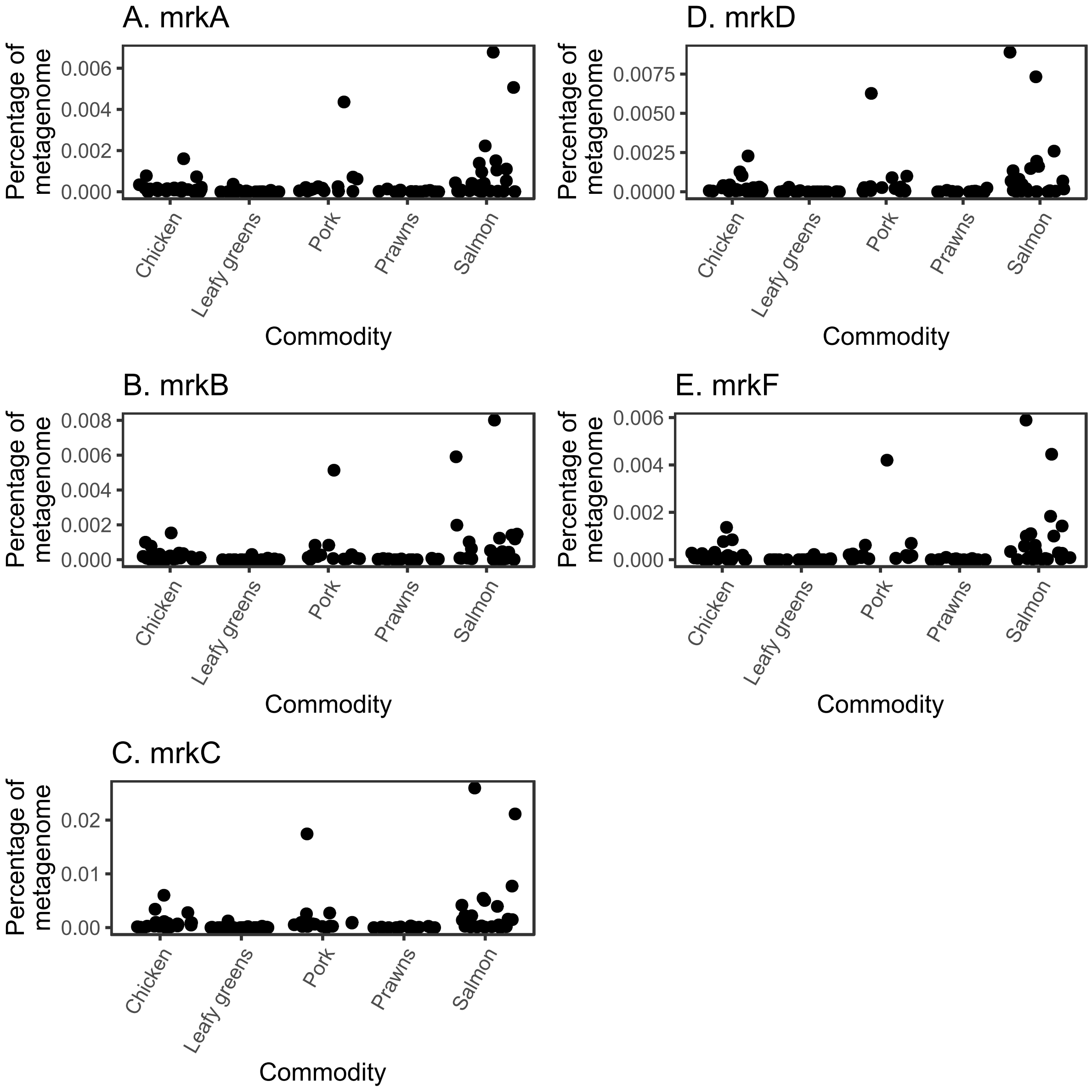

Supplementary figure 8. Percentage of host-removed reads made up of virulence genes that differed that significantly differed amongst food commodities for 106 short-read sequenced food metagenomes that passed QC.

Supplementary table 5. Sensitivity and specificity of the detection of virulence genes associated with pathogens and pathogens cultured from the associated sample.

	Culture positive		Culture negative		Statistics	
Pathogen	Virulence gene positive	Virulence gene negative	Virulence gene positive	Virulence gene negative	Sensitivity	Specificity
Escherichia coli	21	47	4	37	31%	90%
Klebsiella	24	10	67	8	71%	11%
Salmonella	0	3	26	80	0%	75%
Vibrio	2	9	2	29	18%	94%

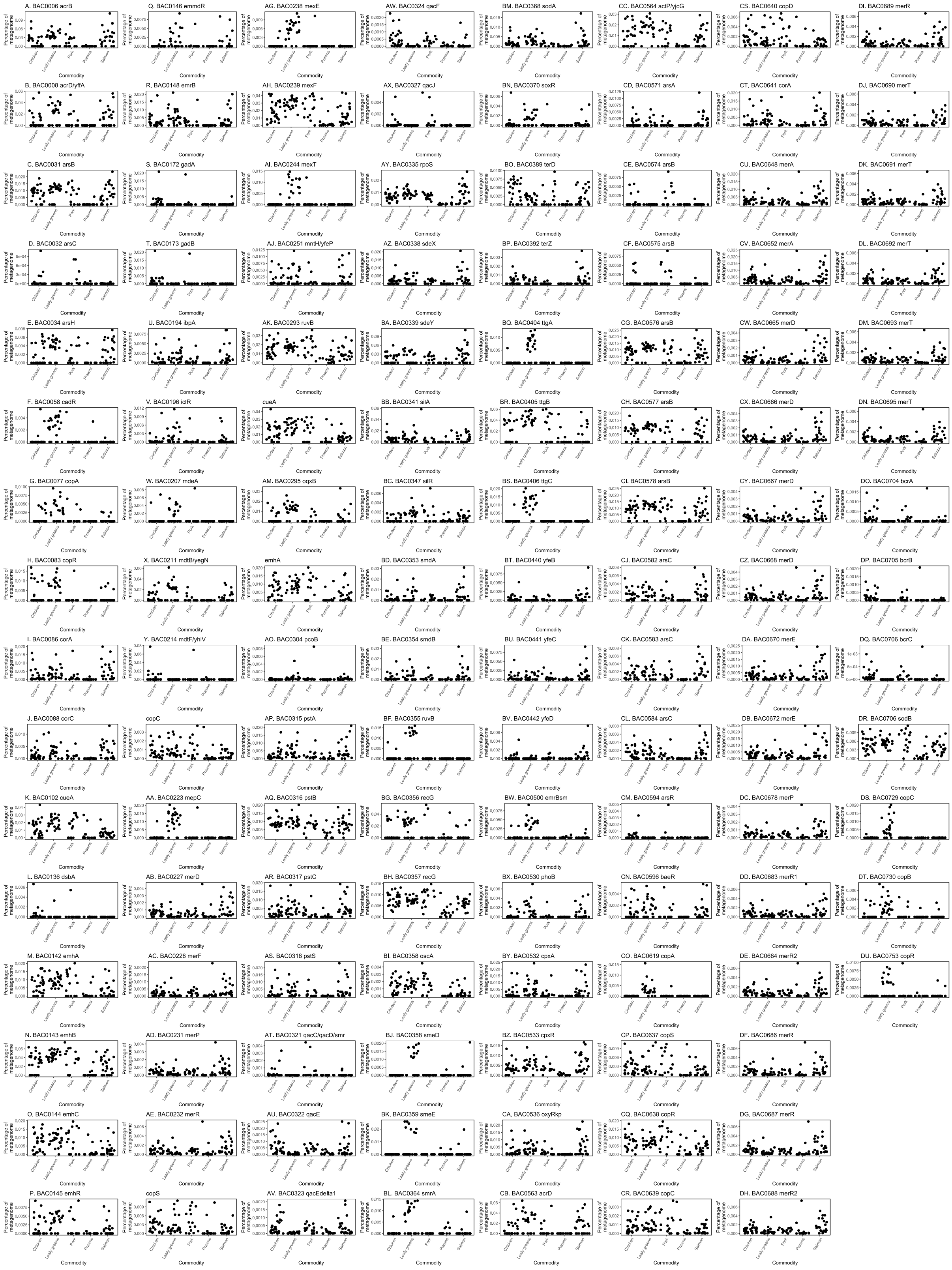

Supplementary figure 9. Percentage of host-removed reads made up of metal-tolerance genes that differed that significantly differed amongst food commodities for 106 short-read sequenced food metagenomes that passed QC.

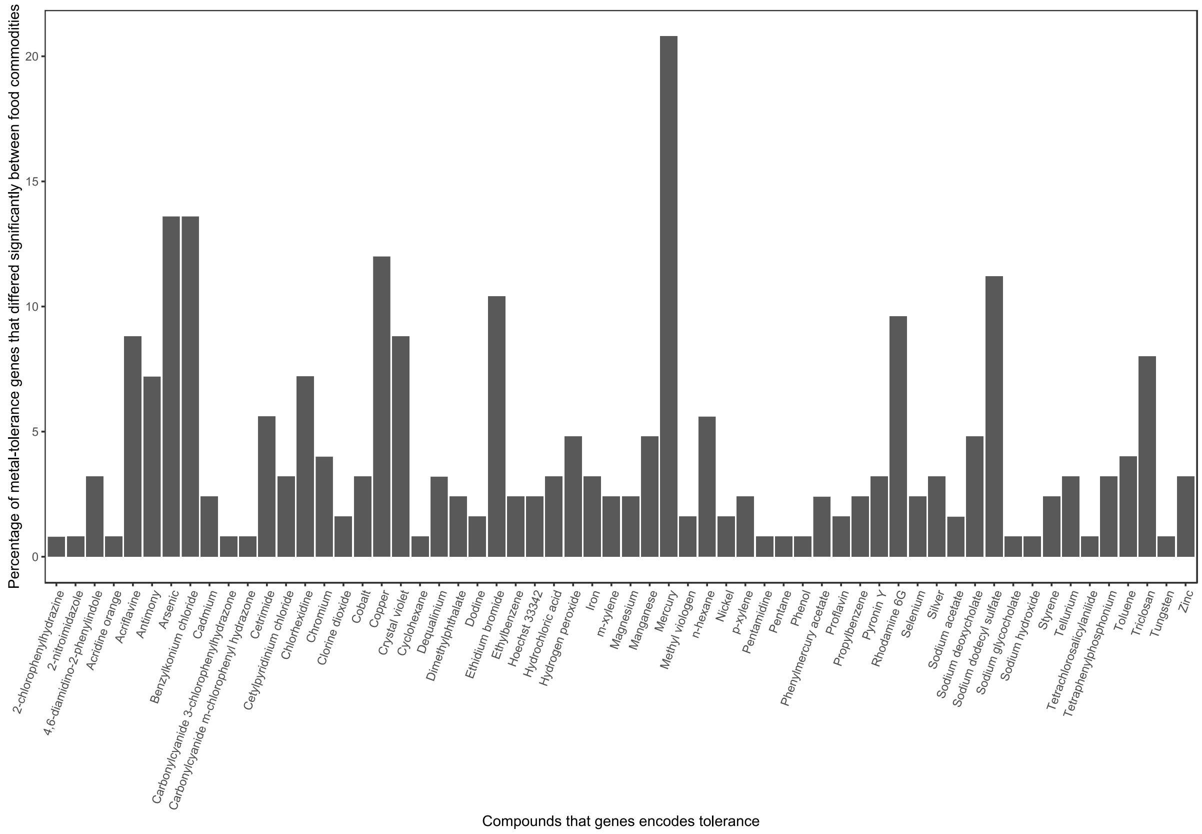

Supplementary figure 10. Percentage of metal-tolerance genes that encode tolerance to compounds amongst those whose presence significantly differed amongst food commodities.

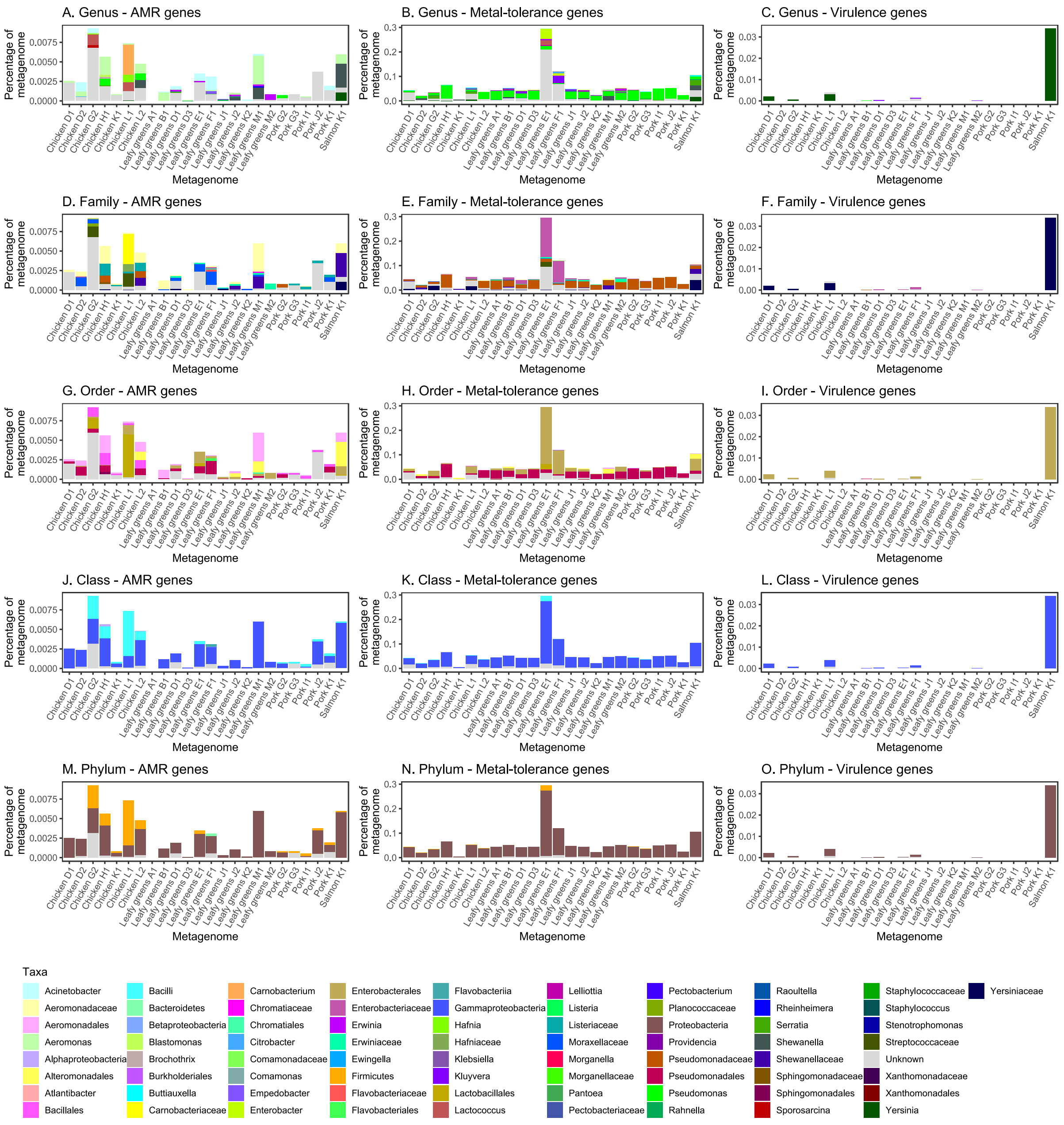

Supplementary figure 11. Percentage of metagenomes made up of antimicrobial resistance (A, D, G, J and M), metal-tolerance (B, E, H, K and N) and virulence (C, F, I, L and O) genes for 24 long-read sequenced metagenomes and coloured by the predicted taxon at the genus (A-C), family (D-F), order (G-I), class (J-L) and phylum (M-O) levels.

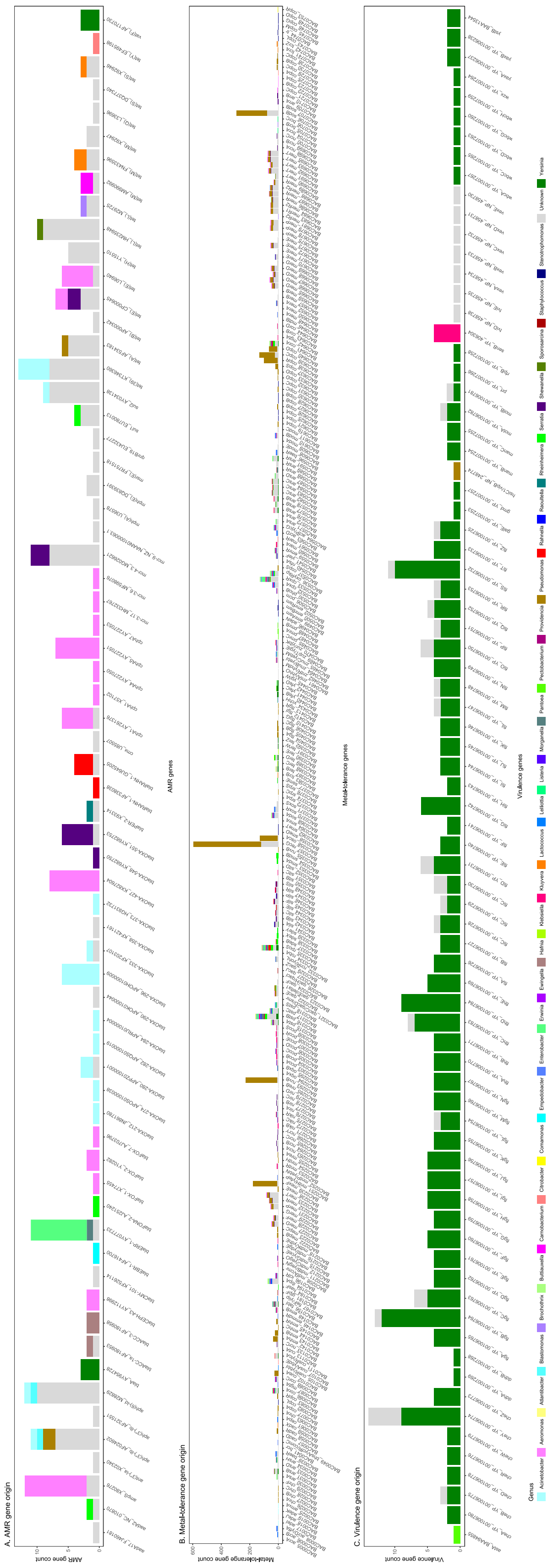

Supplementary figure 12. Number of copies of specific antimicrobial resistance (A), metal-tolerance (B) and virulence (C) genes identified in 24 long-read sequenced metagenomes and coloured by the predicted taxon at the genus level.

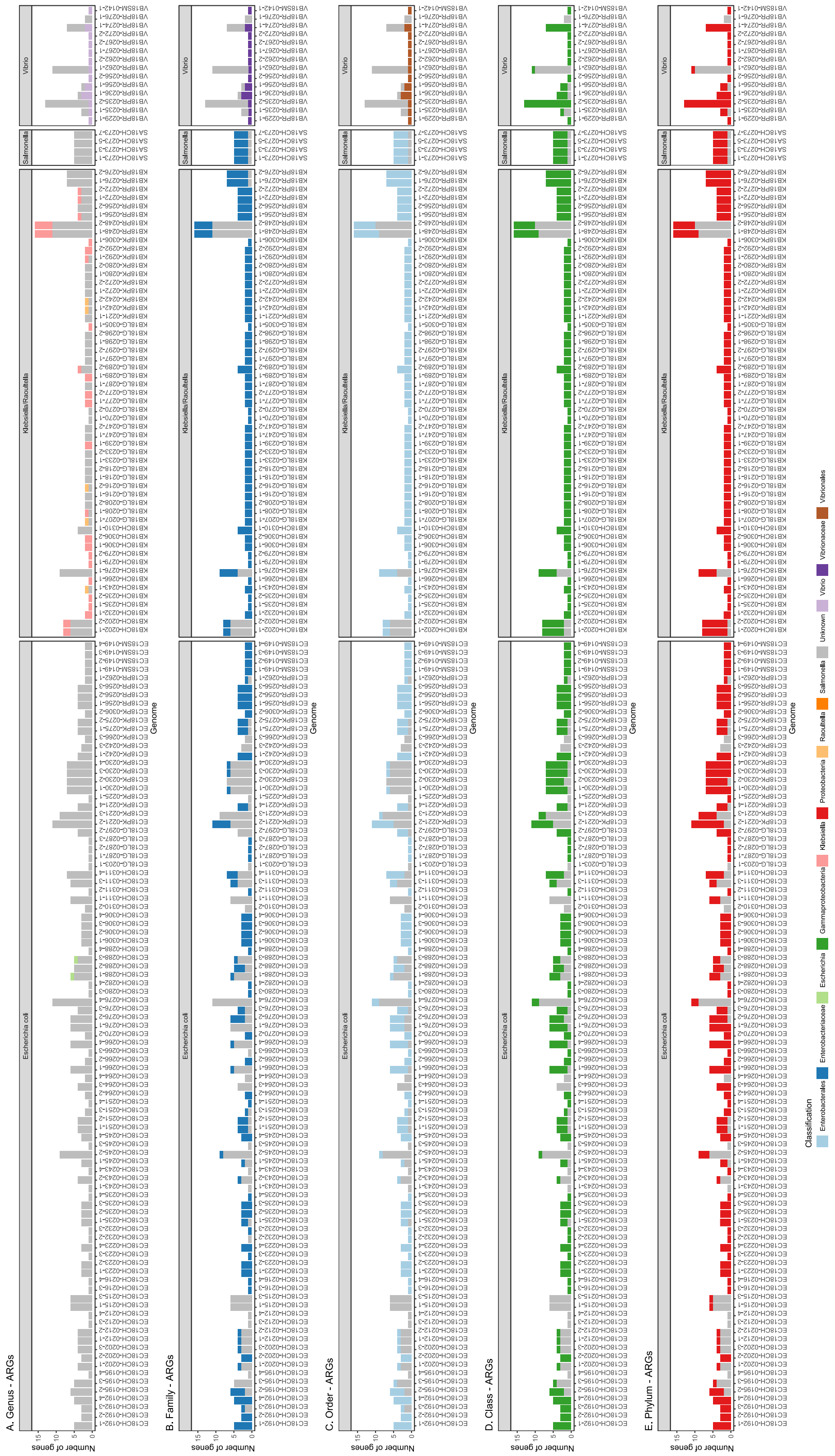

Supplementary figure 13. Number of antimicrobial resistance genes found in 166 genomes containing these types of genes and coloured by the predicted taxon at the genus (A), family (B), order (C), class (D) and phylum (E) levels. Genomes that contained no antimicrobial resistance genes were excluded.

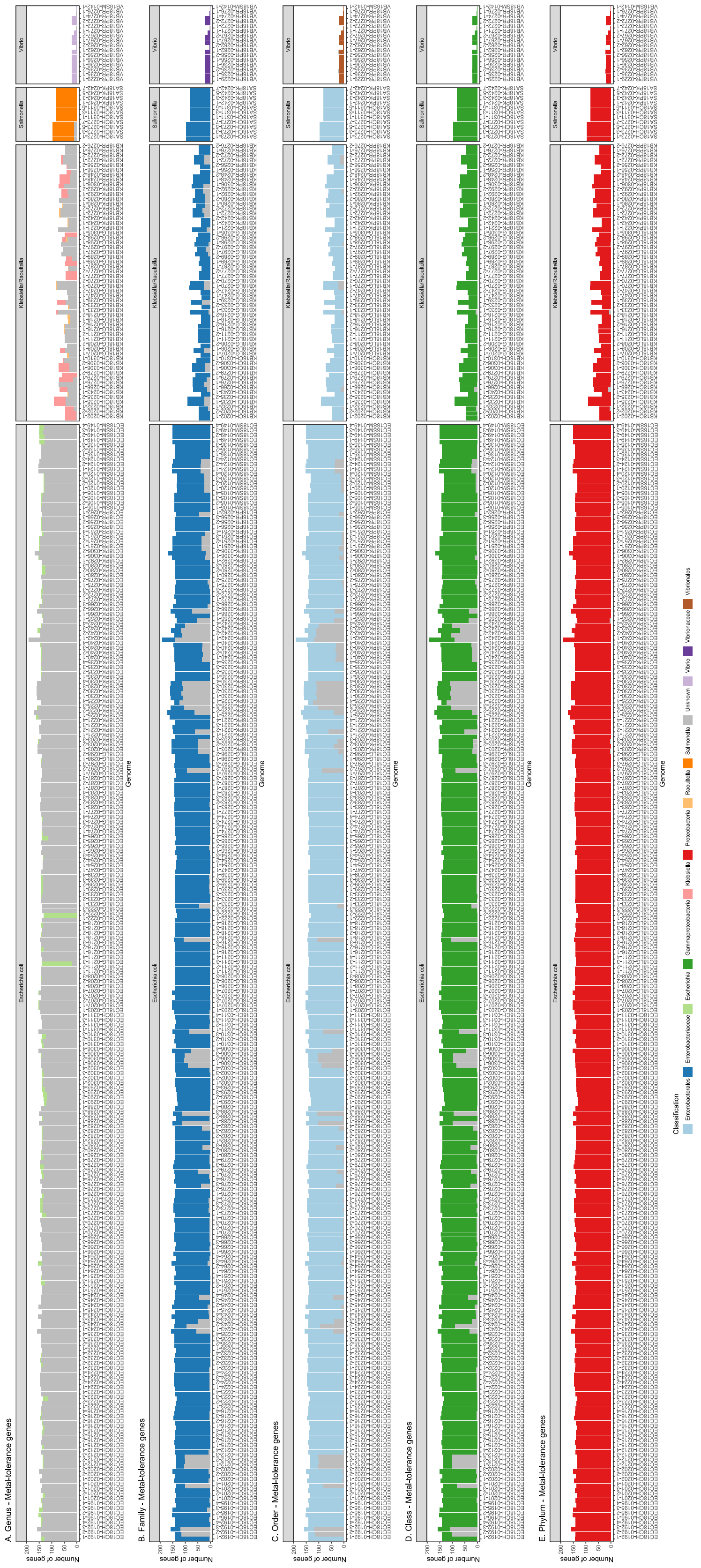

Supplementary figure 14. Number of metal-tolerance genes found in 315 genomes containing these types of genes and coloured by the predicted taxon at the genus (A), family (B), order (C), class (D) and phylum (E) levels. Genomes that contained no metal-tolerance genes were excluded.

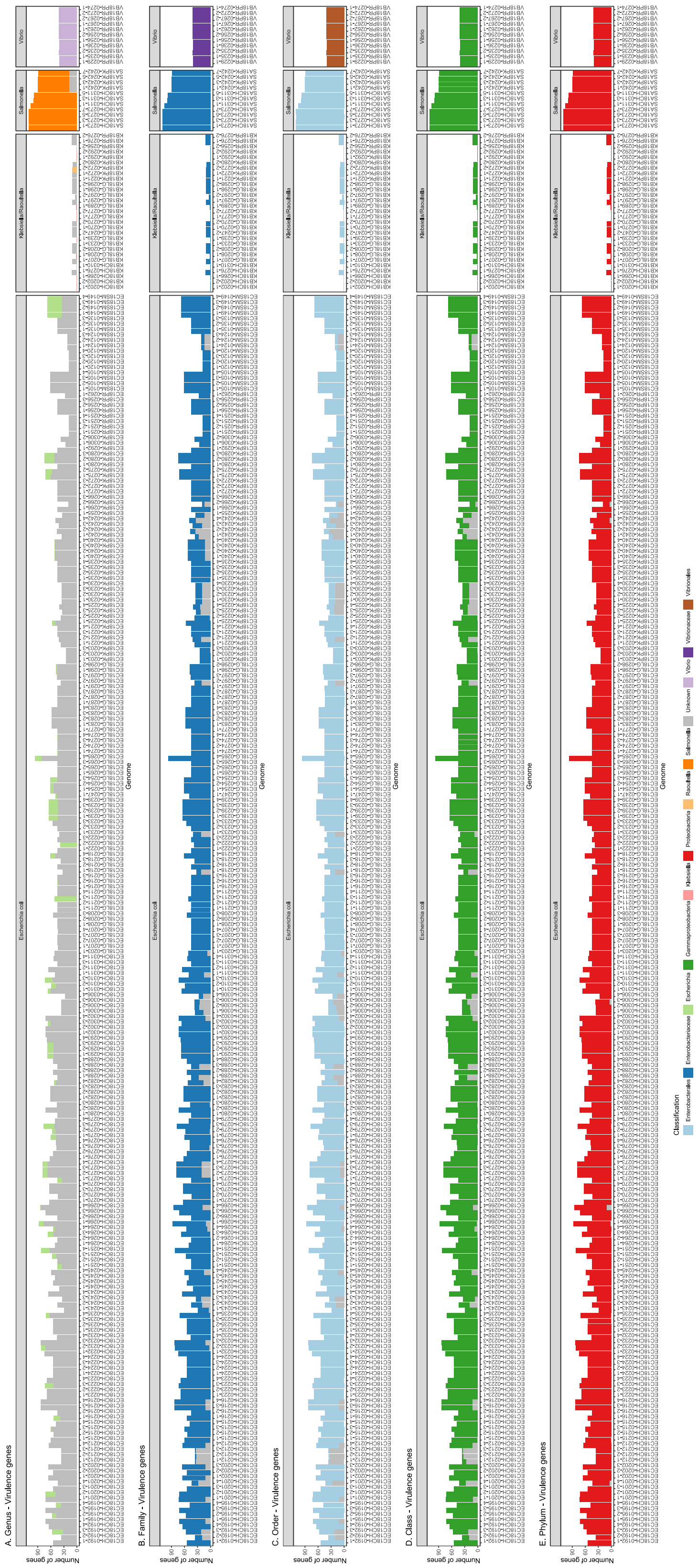

Supplementary figure 15. Number of virulence genes found in 282 genomes containing these types of genes and coloured by the predicted taxon at the genus (A), family (B), order (C), class (D) and phylum (E) levels. Genomes that contained no virulence genes were excluded.
